# Erratum to “Nanotherapeutic System with Effective Microwave Sensitization and Pyroptosis Programming Enable Synergistic Microwave-Immunotherapy in Bladder Cancer”

**DOI:** 10.34133/bmr.0296

**Published:** 2025-12-10

**Authors:** Hao Deng, Jinliang Huang, Ning Gao, Zhi Liu, Zhenglin Yi, Jiatong Xiao, Xin Gao, Chunyu Zhang, Matsika Juliet, Jiao Hu, Jinbo Chen, Xiongbing Zu

**Affiliations:** ^1^ Department of Urology, Xiangya Hospital, Central South University, Changsha, Hunan, China.; ^2^ National Clinical Research Center for Geriatric Disorders, Xiangya Hospital, Central South University, Changsha, China.; ^3^ Department of Urology, The First Affiliate Hospital of Hunan Normal, University (Hunan Provincial People’s Hospital), Changsha, Hunan Province China.; ^4^ Department of Urology, Southwest Hospital, Army Medical University, Chongqing, People’s Republic of China.

In the Research Article titled Nanotherapeutic System with Effective Microwave Sensitization and Pyroptosis Programming Enable Synergistic Microwave-Immunotherapy in Bladder Cancer” [1], the authors identified an inadvertent error in Figs. 3B and 4A, which resulted from operational mistakes during the image merging process. The authors have verified the original data and corrected these figures. The updated versions are provided below and have been incorporated into the online version of the article. Importantly, the authors wish to emphasize that this error was confined solely to the figure assembly stage and does not affect any of the experimental data, results, or scientific conclusions reported in the study. The authors sincerely apologize for this oversight.

**Fig. 3.**
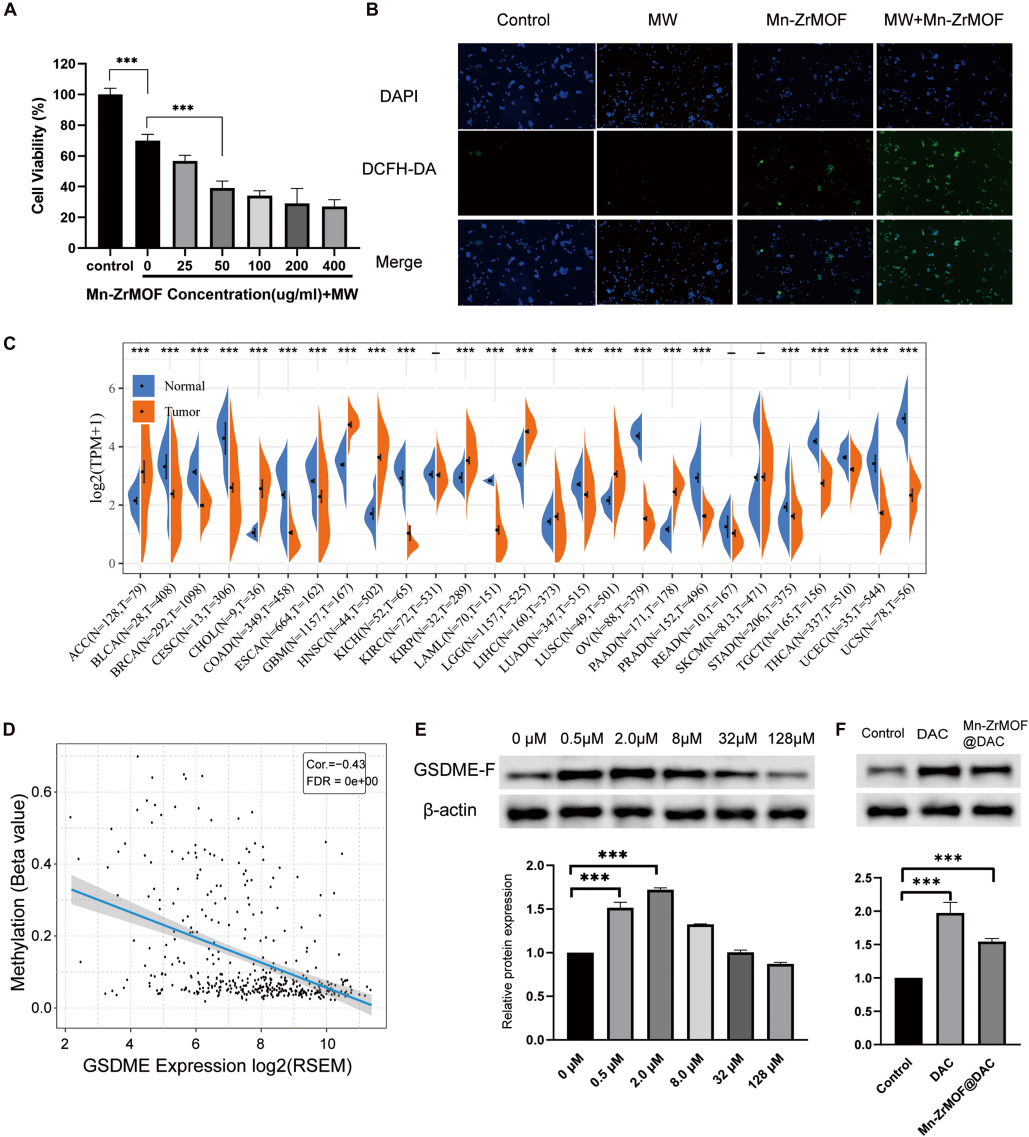
Mn-ZrMOF@DAC inducing ROS production and up-regulating GSDME to achieve antitumor effects. (A) Inhibition of cell viability by different concentration gradients of Mn-ZrMOF nanoparticles under MW radiation (5 W, 5 min). (B) ROS staining results of different treatment groups, where green fluorescence signifies ROS production. (C) Expression of GSDME in various tumors, with low expression of GSDME in tumor tissues of bladder cancer patients. (D) Negative correlation between GSDME expression and methylation level in bladder cancer. (E) Protein expression of GSDME after treatment with different concentrations of DAC in MB49 cells, and (F) protein expression following treatment with DAC-loaded Mn-ZrMOF@DAC.

**Fig. 4.**
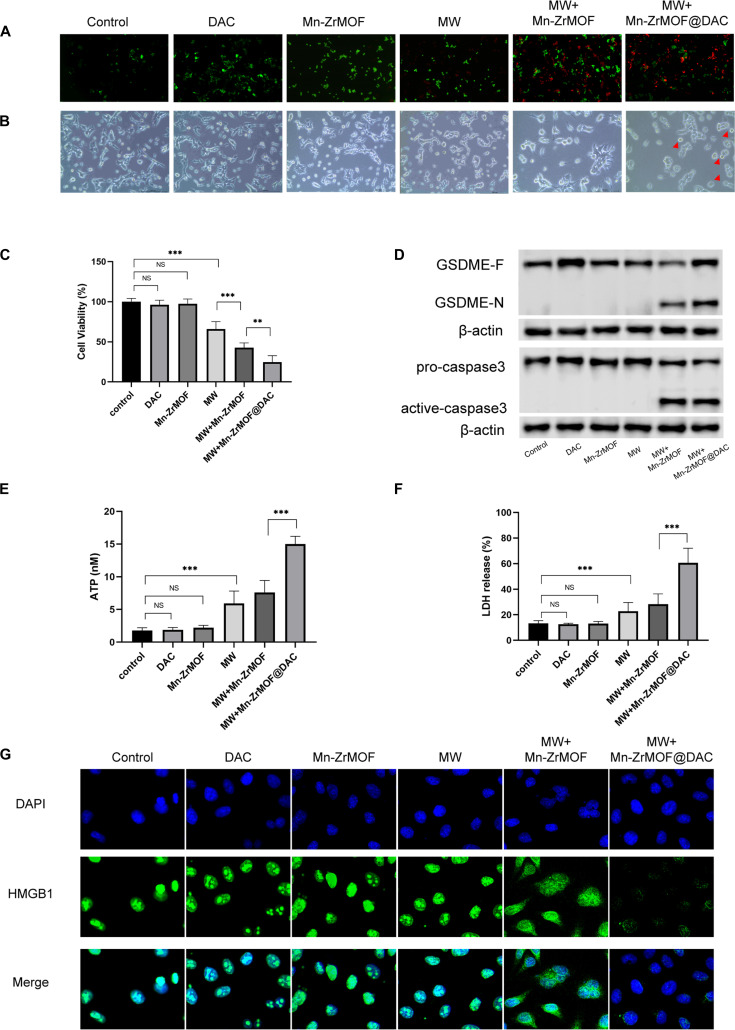
Mn-ZrMOF@DAC inducing MB49 cell pyroptosis by up-regulating GSDME expression. (A) Live/dead staining results of cells from each group. (B) Cellular morphologyunder a light microscope for each group. (C) CCK8 assay results for cell viability in each group. (D) Western blot results for GSDME protein, caspase-3 protein, and their activefragments in each group. (E) ATP release assay results. (F) LDH release assay results. (G) Immunofluorescence results for HMGB1.
