# Supplementary material for: Nanotherapeutic System with Effective Microwave Sensitization and Pyroptosis Programming Enable Synergistic Microwave-Immunotherapy in Bladder Cancer
Source: Biomater Res. 2024 Sep 10;28:0077. doi: 10.34133/bmr.0077 (PMC12237075; doi:10.34133/bmr.0077)
Supplement: 20240910-1 [file bmr.0077.v1.pdf]

Biomaterials Research
A SCIENCE PARTNER JOURNAL

RESEARCH ARTICLE

Nanotherapeutic System with Effective Microwave Sensitization and Pyroptosis Programming Enable Synergistic Microwave-Immunotherapy in Bladder Cancer

Hao Deng[1,2,4†], Jinliang Huang[1,2†], Ning Gao[1], Zhi Liu[1], Zhenglin Yi[1], Jiatong Xiao[1], Xin Gao[1], Chunyu Zhang[1], Matsika Juliet[1], Jiao Hu[1,2*], Jinbo Chen[1,2*], and Xiongbing Zu[1,2,3*]

[1]Department of Urology, Xiangya Hospital, Central South University, Changsha, Hunan, China. [2]National Clinical Research Center for Geriatric Disorders, Xiangya Hospital, Central South University, Changsha, China. [3]Department of Urology, The First Affiliate Hospital of Hunan Normal, University (Hunan Provincial People's Hospital), Changsha, Hunan Province China. [4]Department of Urology, Southwest Hospital, Army Medical University, Chongqing, People's Republic of China.

*Address correspondence to: chenjinbo@csu.edu.cn (J.C.); zuxbxyyy@126.com (X.Z.); hujiao@csu.edu.cn (H.J.)
†These authors contributed equally to this work.

Citation: Deng H, Huang J, Gao N, Liu Z, Yi Z, Xiao J, Gao X, Zhang C, Juliet M, Hu J, et al. Nanotherapeutic System with Effective Microwave Sensitization and Pyroptosis Programming Enable Synergistic Microwave-Immunotherapy in Bladder Cancer. *Biomater. Res.* 2024;28:Article 0077. https://doi.org/10.34133/bmr.0077

Submitted 24 December 2023
Accepted 10 August 2024
Published 10 September 2024

Copyright © 2024 Hao Deng et al. Exclusive licensee Korean Society for Biomaterials, Republic of Korea. No claim to original U.S. Government Works. Distributed under a Creative Commons Attribution License 4.0 (CC BY 4.0).

Currently, the prognosis for patients with advanced bladder cancer remains poor, with only a minority being sensitive to immune checkpoint inhibitors. There is a need to develop additional treatment strategies. Microwave therapy, as a promising approach for some inoperable tumors, still faces challenges such as limited efficacy and high recurrence rates. Additionally, the cell damage and necrosis induced by conventional microwave treatment only act as weak immunostimulatory factors for antitumor immunity, failing to activate effective antitumor immune responses. Recent discoveries have shown that inducing pyroptosis can provide a good opportunity for enhancing systemic immune responses and alleviating immune suppression in cancer therapy. Here, we have developed Mn-ZrMOF@DAC, a microwave-sensitized nanoparticle loaded with the DNA methylation inhibitor decitabine. The Mn-ZrMOF@DAC can enhance the effect of microwave thermal therapy and generate reactive oxygen species under microwave irradiation, causing thermal and oxidative damage to cancer cells. Furthermore, there was an important up-regulation of the key pyroptosis protein GSDME, with a marked increase in pyroptotic cell numbers. In vivo experiments demonstrated that mice injected with Mn-ZrMOF@DAC nanoparticles followed by microwave radiation treatment exhibited potent antitumor effects and enhanced the efficacy of anti-PD-1 therapy. This therapy not only enhanced the efficacy of microwave treatment, exhibiting significant antitumor effects, but also activated antitumor immunity by inducing pyroptosis, thus enhancing the efficacy of immunotherapy for bladder cancer. It holds promise for providing new avenues in the treatment of bladder cancer.

Introduction

Bladder cancer ranks among the top 10 most common cancers worldwide [1]. Despite combined neoadjuvant chemotherapy and surgical treatment, nearly 30% to 40% of patients die within 3 to 4 years post-treatment due to tumor recurrence or progression, with the prognosis for advanced bladder cancer patients remaining poor [2]. Current research has confirmed that cancer immunotherapy, including immune checkpoint blockade, can offer significant survival benefits for patients with advanced bladder cancer. Regrettably, only a minority of patients are sensitive to immune checkpoint inhibitors [3–5]. There are reports of preclinical trials combining tumor immunotherapy with chemotherapy or radiotherapy [6]. However, chemotherapy or radiotherapy inflicts severe damage to the body. Therefore, developing a less toxic therapeutic method to combine with immunotherapy strategies is of great importance.

Microwave (MW) therapy, due to its simplicity of operation, high efficiency, and minimal side effects, is widely considered a promising clinical treatment modality, particularly for patients with inoperable tumors, where it has demonstrated significant survival benefits [7,8]. However, conventional microwave therapies still encounter issues such as suboptimal therapeutic effects and high tumor recurrence rates. Multimodal synergistic therapy

based on multifunctional nanomaterials holds promise for addressing the deficiencies of solo microwave hyperthermia [9]. Recent reports have illustrated a variety of nanomaterials with hollow morphologies and abundant porosity utilized to enhance microwave thermal conversion efficiency, such as layered molybdenum disulfide (MoS_2) nanoflowers, popcorn-like Zr-MOF nanoparticles, and calcium alginate hydrogel [10–13]. The combination of microwave hyperthermia and microwave dynamic therapy, generating both thermal effects and reactive oxygen species (ROS) upon microwave activation, appears to be a more attractive synergistic approach for improving antitumor efficacy [14]. Nonetheless, the complex biological characteristics of tumors can sometimes affect the efficacy of microwave hyperthermia. The tumor cell damage and necrosis induced by microwave treatment have always been merely weak immunostimulatory factors for antitumor immunity, insufficient to activate effective antitumor immune responses [15]. The recent discovery that inducing pyroptosis can provide good opportunities for promoting systemic immune responses and alleviating immune suppression in cancer therapy suggests that converting cell death during microwave treatment into immunogenic pyroptosis could effectively resolve this issue [16,17].

Pyroptosis is a highly inflammatory form of programmed cell death mediated by the gasdermin (GSDM) protein family, which has garnered significant attention in recent years [16]. GSDM proteins can be activated by caspases or granzymes, and active GSDM proteins lead to cell membrane perforation and subsequent pyroptosis. During this process, a multitude of cytokines and danger-associated molecular patterns (DAMPs) are released, enhancing the infiltration of immune cells such as CD8+ T cells into the tumor tissue, further amplifying inflammatory responses, and activating the body's antitumor immunity [18,19]. Multiple studies have shown that inducing tumor cell pyroptosis has the potential to enhance the efficacy of immunotherapy [20,21]. Notably, within the GSDM protein family, the GSDME protein can be cleaved and activated by caspase-3, initiating an alternative pathway for pyroptosis [22]. This implies that microwave treatment can be used to generate a significant quantity of ROS in tumor cells to activate caspase-3, thereby triggering GSDME protein-based pyroptosis. Unfortunately, the expression of GSDME protein, closely associated with the methylation of the GSDME gene, is often much lower in most tumor cells compared to normal cells, which may be a potential mechanism by which tumor cells avoid GSDME-mediated pyroptosis, making it difficult to activate the pyroptosis-induced antitumor immune pathway during treatment [23,24]. Nevertheless, advances in epigenetic therapies now offer hope that DNA methyltransferase (DNMT) inhibitors can be used to reverse DNA methylation modifications and restore normal protein expression [25]. Decitabine (DAC), one of the most commonly used DNMT inhibitors, has been approved by the U.S. Food and Drug Administration for the treatment of hematologic conditions such as myelodysplastic syndromes and can also be employed to increase the expression of methylated genes [26].

In this study, utilizing The Cancer Genome Atlas (TCGA) database, we found GSDME to be significantly down-regulated in bladder, and that GSDME expression is negatively regulated by DNA methylation. Furthermore, low concentrations of DAC significantly up-regulated GSDME expression. Then, we found that Mn-ZrMOF nanoparticles can act as MW sensitizers and generating a substantial quantity of hydroxyl radicals (•OH), promoting the thermal effects of MW radiation. Thus, we designed a microwave-sensitized nanoparticle loaded with the DNA methylation inhibitor DAC, referred to as Mn-ZrMOF@DAC, which was found to promote the thermal effects of microwave radiation and generate ROS under microwave stimulation, causing thermal and oxidative damage to cancer cells. Furthermore, there was a significant up-regulation of the key pyroptosis protein GSDME in bladder cancer cells, with a marked increase in pyroptotic cell numbers. In vivo experiments demonstrated that mice injected with Mn-ZrMOF@DAC nanoparticles followed by microwave radiation treatment exhibited potent antitumor effects and enhanced the efficacy of anti-PD-1 therapy. The specific mechanism is illustrated in Fig. 1.

Materials and Methods

Synthesis of Mn-ZrMOF

Mn-ZrMOF was synthesized according to the method described in the literature [14]. Weigh out 13.4 mg of zirconium tetrachloride, 9.5 mg of terephthalic acid, and 7.5 mg of polyvinylpyrrolidone and placed them in a 20-ml glass vial. To this, 12.45 g of *N*,*N*-dimethylformamide (DMF) was added, and the mixture was sonicated for 5 min to ensure complete dissolution. Then, 2.3 mg of manganese(II) chloride tetrahydrate was added, and the resulting mixture was homogenized to form a uniform solution. Subsequently, the solution was transferred to a Teflon-lined stainless steel autoclave and placed in an oven at 120 °C for 10 h. After cooling to room temperature, the mixture was centrifuged at 4,000 rpm for 15 min, the supernatant was discarded, and the precipitate was washed 3 times with DMF and ethanol. Finally, the washed precipitate was dried in a vacuum oven at 60 °C to obtain Mn-ZrMOF powder.

Synthesis of Mn-ZrMOF@DAC

Mn-ZrMOF (10 mg) and 5 mg of DAC were placed in a 20-ml glass vial, to which 10 ml of anhydrous ethanol was added and stirred at room temperature for 72 h. Afterward, the mixture was centrifuged at 8,000 rpm for 15 min, and the supernatant and precipitate were collected. The drug loading of Mn-ZrMOF@DAC was determined using an ultraviolet (UV)-visible spectrophotometer by analyzing the collected supernatant. The collected precipitate was dried in a vacuum oven at room temperature to obtain Mn-ZrMOF@DAC powder samples, which were stored at −20 °C.

Drug release profile of Mn-ZrMOF@DAC

One milliliter of 3 mg/ml Mn-ZrMOF@DAC solution was placed into a sealed dialysis membrane, which was then immersed in 20 ml of PBS solution at pH 5.5 and pH 7.4, respectively, and placed in a constant temperature shaking incubator. At predetermined time points, the dialysis liquid was sampled for UV-vis absorption spectral measurement.

Characterization of Mn-ZrMOF and Mn-ZrMOF@DAC

The morphological structure of Mn-ZrMOF nanoparticles was observed using scanning electron microscopy (SEM) and transmission electron microscopy (TEM). The hydrated particle size distribution and zeta potential of Mn-ZrMOF and Mn-ZrMOF@DAC were measured using a dynamic light scattering (DLS) and Zeta potential analyzer.

Microwave heat sensitization performance evaluation of Mn-ZrMOF: First, 1.5 ml of different solutions was prepared in cuvettes (water, water+10 mg/ml Mn-ZrMOF, 0.9% NaCl,

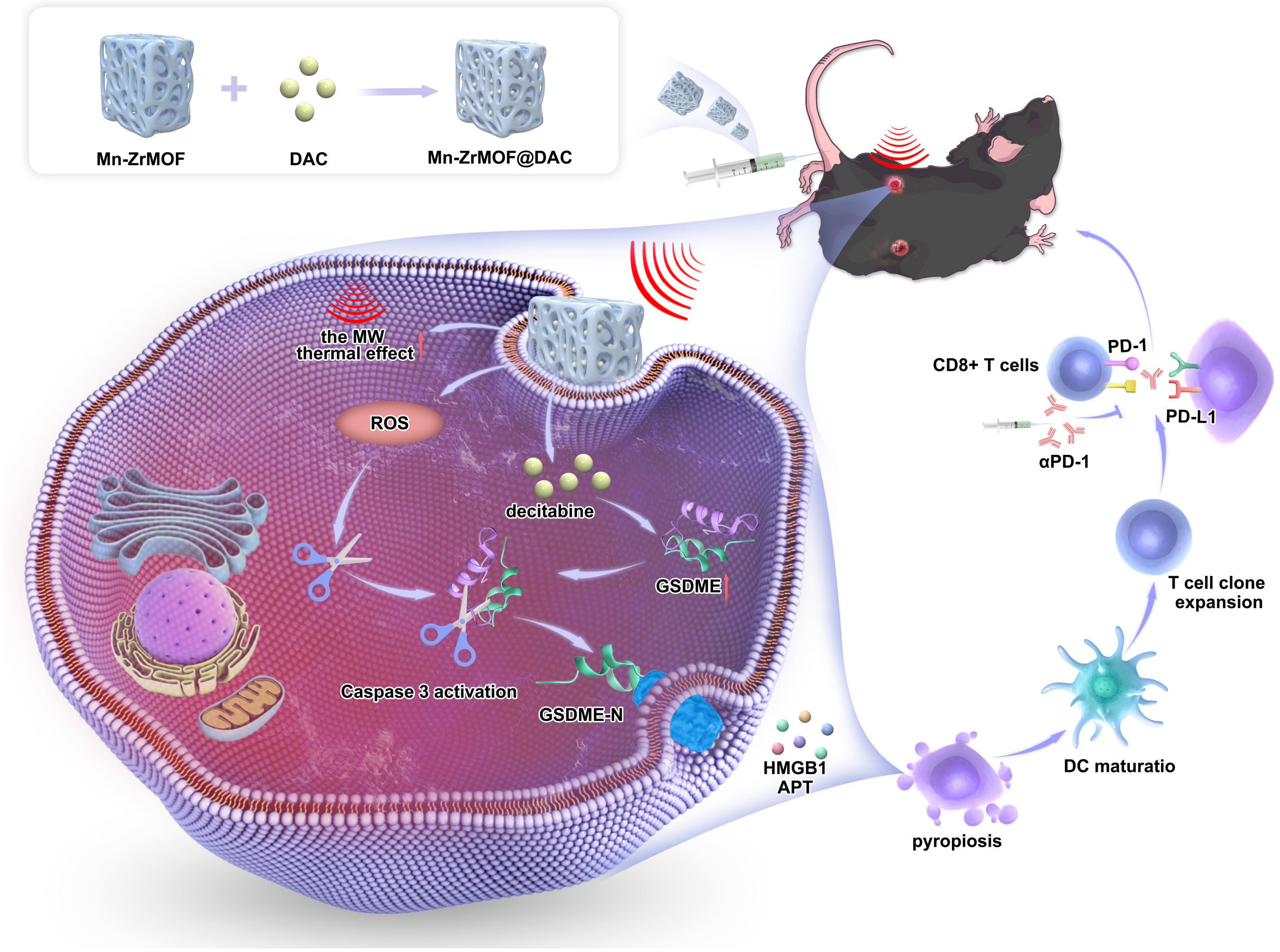

Fig. 1. Schematic diagram of the mechanism.

0.9% NaCl+5 mg/ml Mn-ZrMOF, 0.9% NaCl+10 mg/ml Mn-ZrMOF, and 0.9% NaCl+20 mg/ml Mn-ZrMOF). Then, MW irradiation (5 W, 5 min) was applied to each group using a microwave therapy device. During this period, an infrared thermal imager was used to monitor temperature changes in real time, and temperature rise curves were plotted.

TMB assay for peroxidase-like activity

The generation of •OH was observed using 3,3′,5,5′-tetramethylbenzidine (TMB): TMB can be oxidized by hydroxyl radicals (•OH) to form blue oxidized TMB, which exhibits a characteristic absorption peak at 652 nm. TMB chromogenic liquid, H_2O_2, and Mn-ZrMOF nanoparticle aqueous solution were added to 2 centrifuge tubes, one of which was treated with MW (5 W) and the other served as a control. At various time points, UV absorption measurements were taken for the supernatants of both groups, and results were normalized for analysis.

Cell culture and animals

MB49 cells were purchased from Beina Biotechnology Co., Ltd. and were cultured in RPMI-1640 medium supplemented with 10% fetal bovine serum and 1% penicillin/streptomycin, with media changes every 2 days, or upon reaching 80% to 90% confluence in the culture flask, at which point cells were passaged. C57 mice used in this study were sourced from the Animal Experiment Center of Central South University. Animal care and experimentation were strictly conducted in accordance with the institutional guidelines for animal experiments, and the study protocol was approved by the Ethics Committee of the Animal Experiment Center at Central South University.

CCK-8 assay for cell viability

MB49 cells were prepared into cell suspensions and seeded in a 96-well plate at 2,000 cells per well. After culturing for 12 h in a cell incubator, different gradients of Mn-ZrMOF nanoparticles were added according to the experimental plan and treated for 12 h, with groups requiring microwave irradiation being treated with MW (5 W, 5 min). After treatment, cells were further cultured for 24 h. The culture medium containing intervention drugs was removed by pipetting, and cells were washed twice before adding serum-free medium. Each well received 10 μl of CCK-8 reagent. The culture plate was incubated for 1 h at 37 °C in a CO_2 incubator, and the optical density (OD) at 450 nm was measured using an automatic microplate reader.

ROS detection assay

MB49 cells were seeded at 2×10^5 cells per well in a 6-well plate and cultured for 12 h. Groups were treated according to the

experimental plan (control group, MW group, Mn-ZrMOF group, and Mn-ZrMOF+MW group). After 12 h of adding Mn-ZrMOF nanoparticles or control solvent, DAPI dye (final concentration 1 μg/ml) was added and incubated for another 12 h, with MW treatment groups receiving additional MW (5 W, 5 min) treatment. After 15 min, the culture medium containing materials was discarded, and cells were washed 3 times with PBS. Then, DCFH-DA probe (final concentration, 20 μmol/l) and serum-free medium were added and incubated at 37 °C for 15 min. Subsequently, the medium containing DCFH-DA was discarded; cells were washed 3 times with PBS and observed and photographed under a fluorescence microscope.

Live/dead cell staining assay

MB49 cells were seeded at 2×10^5 cells per well in a 6-well plate and cultured for 12 h. Groups were treated according to the experimental plan (control group, DAC group, Mn-ZrMOF group, MW group, Mn-ZrMOF+MW group, and Mn-ZrMOF@DAC+MW group), with respective additions of control solvent, DAC, Mn-ZrMOF, or Mn-ZrMOF@DAC (concentration of DAC was 3.3 μg/ml, concentration of Mn-ZrMOF was 50 μg/ml, and concentration of Mn-ZrMOF@DAC was 58.4 μg/ml) and incubated for 12 h, with MW treatment groups receiving additional MW (5 W, 5 min) treatment. After 12 h post-treatment, the culture medium was aspirated, and cells were gently washed twice with PBS buffer. Pre-prepared calcein-AM and propidium iodide (PI) working solution were added and thoroughly mixed, followed by a 10-min incubation. Cells were washed twice with PBS buffer. The staining of AM and PI was observed and photographed under a fluorescence microscope.

Western blot analysis

Western blot analysis was conducted as previously reported. After group treatments, mouse tumor cells were lysed using radioimmunoprecipitation assay reagent containing a complete protease inhibitor mixture and phosphatase inhibitors. Proteins were separated using 10% SDS-polyacrylamide gel electrophoresis and transferred onto polyvinylidene difluoride membranes. Following incubation with primary antibodies, corresponding secondary antibodies were used, and protein bands were visualized using ECL detection reagent.

LDH release assay

MB49 cells were seeded at 2,000 cells per well in a 96-well plate and cultured for 12 h. Groups were treated according to the experimental plan (control group, DAC group, Mn-ZrMOF group, MW group, Mn-ZrMOF+MW group, and Mn-ZrMOF@DAC+MW group), with respective additions of control solvent, DAC, Mn-ZrMOF, or Mn-ZrMOF@DAC (concentration of DAC was 3.3 μg/ml, concentration of Mn-ZrMOF was 50 μg/ml, and concentration of Mn-ZrMOF@DAC was 58.4 μg/ml) and incubated for 12 h, with the MW treatment groups receiving additional MW (5 W, 5 min) treatment. After another 12 h of incubation post-treatment, the supernatant was transferred to a new 96-well plate, and the level of lactate dehydrogenase (LDH) in the cell culture supernatant was detected using an LDH release assay kit.

ATP release assay

MB49 cells were seeded at 2×10^5 cells per well in a 6-well plate and cultured for 12 h. Groups were treated according to the experimental plan (control group, DAC group, Mn-ZrMOF group, MW group, Mn-ZrMOF+MW group, and Mn-ZrMOF@DAC+MW group), with respective additions of control solvent, DAC, Mn-ZrMOF, or Mn-ZrMOF@DAC (concentration of DAC was 3.3 μg/ml, concentration of Mn-ZrMOF was 50 μg/ml, and concentration of Mn-ZrMOF@DAC was 58.4 μg/ml) and incubated for 12 h, with the MW treatment groups receiving additional MW (5 W, 5 min) treatment. After another 12 h of incubation post-treatment, the supernatant from each group was collected, and the adenosine triphosphate (ATP) concentration in the supernatant was measured using an ATP assay kit.

Immunofluorescence for cells

MB49 cells were seeded at 2×10^4 cells per well in a 24-well plate and cultured for 12 h. Groups were treated according to the experimental plan (control group, DAC group, Mn-ZrMOF group, MW group, Mn-ZrMOF+MW group, and Mn-ZrMOF@DAC+MW group), with respective additions of control solvent, DAC, Mn-ZrMOF, or Mn-ZrMOF@DAC (concentration of DAC was 3.3 μg/ml, concentration of Mn-ZrMOF was 50 μg/ml, and concentration of Mn-ZrMOF@DAC was 58.4 μg/ml) and incubated for 12 h, with the MW treatment groups receiving additional MW (5 W, 5 min) treatment. After another 12 h of incubation post-treatment, cells were fixed with 4% paraformaldehyde and permeabilized with 0.1% Triton X-100. Cells were then incubated with anti-HMGB1 antibodies and FITC-conjugated antibodies. Cells were counterstained with DAPI. Fluorescence microscopy was used to observe and capture images of the experiment, where green fluorescence represents HMGB1 and blue fluorescence represents the cell nucleus.

Establishment of a subcutaneous tumor model in C57 mice

Six-week-old C57 mice were subcutaneously injected with 1×10^5 MB49 cells in the right flank. When the subcutaneous tumors reached a volume of approximately 100 mm^3, the mice were randomly divided into 6 groups (5 mice per group) for corresponding treatments: (a) control group; (b) DAC group; (c) Mn-ZrMOF group; (d) MW group; (e) Mn-ZrMOF+MW group; and (f) Mn-ZrMOF@DAC+MW group. Different groups received intratumoral injections of PBS solution, DAC solution, Mn-ZrMOF solution, or Mn-ZrMOF@DAC solution; concentration of DAC was 1.3 mg/kg, concentration of Mn-ZrMOF was 20 mg/kg, and concentration of Mn-ZrMOF@DAC was 23.36 mg/kg. MW treatment groups received MW (5 W, 5 min) treatment 12 h after drug administration. Tumor dimensions and body weight were measured and recorded every 2 days. The tumor volume V was calculated using the formula $V = 0.5 \times L \times W2$, where L is the longest diameter of the tumor and W is the shortest diameter. After 12 days of treatment, mice were euthanized, and the tumors and vital organs were collected for subsequent experiments.

The detection of TNF-α and IFN-γ levels in mouse serum

Blood samples were collected from each group of mice and allowed to naturally solidify at room temperature for 10 to 20 min. The samples were then centrifuged at 4 °C and 2,000 rpm for approximately 20 min. The supernatant was carefully collected using a pipette after centrifugation. The detection of TNF-α and IFN-γ in the serum of each group was performed using a reagent kit.

Tumor tissue was dissolved into a homogenate and subsequently digested with enzymes in a moist incubator at 37 °C and 5% CO_2

for 1 h. The resulting single-cell suspension was filtered and collected using a cell sieve. Following the utilization of red blood cell lysate, viability staining was conducted, followed by the addition of anti-mouse CD16/CD32 monoclonal antibody for a duration of 10 min to facilitate blocking. Subsequently, a fluorescent labeled antibody reagent was added and gently mixed (PE anti-mouse CD11c, PerCP/Cyanine5.5 antimouse CD4, PE/Cyanine7 anti-mouse CD86, APC anti-mouse CD80, Alexa Fluor 700 anti-mouse CD8a, APC/Fire 750 anti-mouse CD45, and Brilliant Violet 421 anti-mouse CD3), and the reaction was allowed to proceed under dark conditions at a temperature of 4 °C for a duration of 30 min. Following the necessary washing and resuspension steps, the sample was subjected to detection using a flow cytometer and subsequently analyzed using FlowJo (Tree Star).

Statistics

We utilized Microsoft Excel for data management and analysis, and GraphPad Prism 8 for statistical analysis. Measured data were expressed as mean and standard deviation. Groups were compared using the 2-tailed Student's *t* test or one-way analysis of variance. The differences were considered significant based on the obtained *P* values: $*P < 0.05$, $**P < 0.01$, $***P < 0.001$.

Results

Physicochemical characterization of Mn-ZrMOF@DAC

The Mn-ZrMOF was successfully synthesized via a one-pot hydrothermal method in this study. The powder x-ray diffraction (PXRD) pattern of Mn-ZrMOF nanoparticles displayed diffraction peaks that overlapped well with the simulated pattern of UiO-66 (Zr-MOF), indicating that Mn doping did not alter the fundamental structure of UiO-66 (Zr-MOF) (Fig. 2A). SEM images revealed that the Mn-ZrMOF nanoparticles prepared in this study were uniform nanocubes with an average size of 60±5 nm. When DAC is loaded, its cube shape and size remain relatively unchanged, and DAC is adsorbed within the pores of Mn-ZrMOF without significantly impacting its structural integrity (Fig. 2B). DLS measurements showed that the Mn-ZrMOF nanoparticles had an average hydrodynamic diameter of approximately 161.1 nm with a polydispersity index (PDI) of 0.209. After loading with DAC, there was no change in the morphology of Mn-ZrMOF@DAC, and DLS results revealed an average hydrated particle size of 192.3 nm and a PDI of 0.301 for Mn-ZrMOF@DAC (Fig. 2C). The zeta potential changed from 11.2 mV to −14.6 mV after drug loading (Fig. 2D). These experimental results demonstrate the successful preparation of a stable nanoparticle material, Mn-ZrMOF@DAC, loaded with DAC. DAC exhibited UV absorption at approximately 225 nm, and the loading efficiency of DAC in Mn-ZrMOF@DAC nanoparticles was 16.85%, with in vitro release curves showing that, in acidic conditions, both the release rate and maximum release of DAC were significantly higher than in neutral conditions. The 24-h release rates for the nanomaterial Mn-ZrMOF@DAC were determined to be 51.17% (pH 5.5), 42.14% (pH 6.0), and 20.27% (pH 7.4). As we know, the pH in the tumor microenvironment (TME) is close to 6.0 (Fig. S1). Furthermore, the hydrated particle size of the prepared nanomaterial was analyzed in various solutions and temperatures, revealing no significant differences and indicating a certain level of stability for the nanoparticles (Fig. S2A to C).

MW heating experiment and dynamic study of Mn-ZrMOF nanocubes

The principle behind microwave (MW) radiation heating is the rapid oscillation of polar molecules such as water or ions under microwave exposure, resulting in frictional heat [27]. We conducted MW radiation experiments (5 W, 5 min) in several solutions and plotted their temperature curves. As shown in Fig. 2E, after MW radiation (5 W, 5min), no significant temperature change was observed in pure water or in water solutions containing only Mn-ZrMOF nanoparticles. However, the temperature of a 0.9% NaCl solution rapidly rose to 43 °C, indicating that ions play a significant role in the thermal effects of MW radiation. Additionally, under the same conditions, the rate of temperature increase in a 0.9% NaCl solution with added Mn-ZrMOF nanoparticles was significantly enhanced, and as the concentration of Mn-ZrMOF nanoparticles increased, so did the rate of temperature rise. This suggests that Mn-ZrMOF nanoparticles can promote the conversion of MW energy into thermal energy, enhancing the microwave thermal effect. This enhancement is likely due to the large specific surface area and abundant porosity of the Mn-ZrMOF nanoparticles, which can absorb and accommodate a rich array of ions. In such a scenario, when subjected to MW radiation, ions undergo intense inelastic collisions within a confined area due to the presence of Mn-ZrMOF nanoparticles, resulting in the generation of a substantial amount of heat. By contrast, in a 0.9% NaCl solution without Mn-ZrMOF nanoparticles, the collision movement of ions in free space has a much lower collision frequency, leading to much less frictional heat. Therefore, Mn-ZrMOF nanoparticles can act as MW sensitizers, promoting the thermal effects of MW radiation.

In order to exclude the influence of temperature on the generation of *OH, we used pure water instead of 0.9% NaCl for the test. Upon adding TMB and H_2O_2 to the aqueous solution of Mn-ZrMOF nanoparticles and measuring the UV absorption of the supernatant after the reaction, the results showed a characteristic absorption peak at 652 nm in the absence of microwave radiation, indicating that Mn-ZrMOF nanoparticles catalyze a minor amount of H_2O_2, producing a small quantity of •OH (Fig. S2D). Under microwave radiation, the maximum absorbance significantly increased (Fig. S2E and Fig. 2F), suggesting that MW enhances the peroxidase-like catalytic activity of Mn-ZrMOF nanoparticles, catalyzing H_2O_2 to generate a large amount of •OH. The peroxidase-like activity of Mn-ZrMOF nanoparticles may originate from the doping of the transition metal Mn. Exogenous microwave stimulation resulting in localized high temperatures and inducing efficient electron transfer can accelerate the catalysis of hydrogen peroxide (H_2O_2,) undergoing Fenton-like reactions, generating a substantial quantity of hydroxyl radicals (•OH).

Antitumor effects of Mn-ZrMOF through induction of ROS in synergy with microwave treatment

Initially, the study utilized the CCK-8 cell viability assay to demonstrate that Mn-ZrMOF nanoparticles possess good biocompatibility, as they did not inflict damage on the cells by themselves (Fig. S3A). However, after treating different groups of MB49 cells incubated with varying concentrations of Mn-ZrMOF nanoparticles with microwaves (MW) (5.0 W, 5 min), it was observed that the cell viability of the MW alone group only dropped to 70.2%±4.5%. As the concentration of Mn-ZrMOF nanoparticles increased, the viability of the mouse bladder cancer cells MB49 progressively

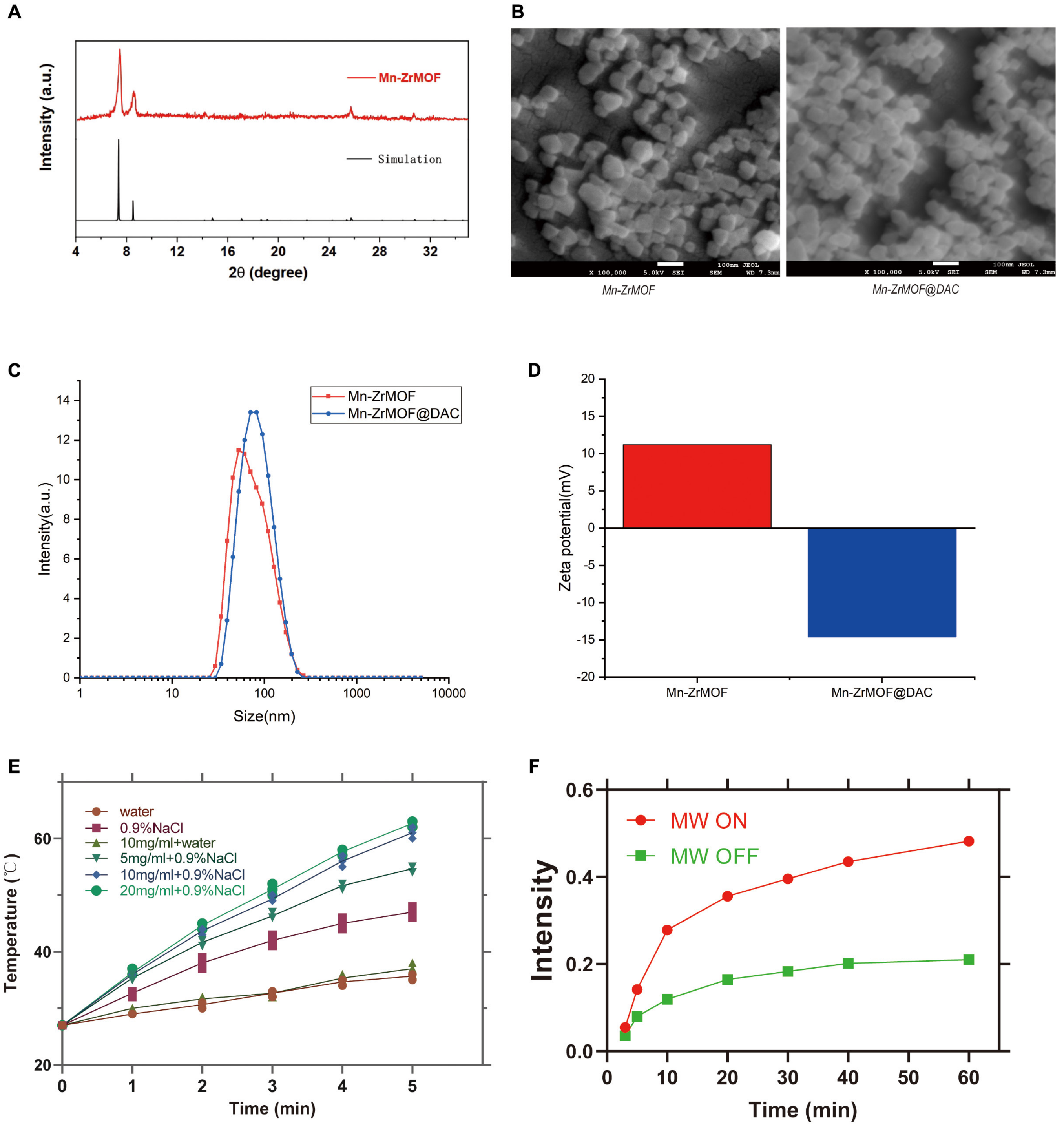

Fig. 2. Characterization of Mn-ZrMOF nanoparticles. (A) SEM image of Mn-ZrMOF nanoparticles. (B) XRD pattern of Mn-ZrMOF nanoparticles. (C) DLS measurements of Mn-ZrMOF nanoparticles before and after drug loading. (D) Zeta potential of Mn-ZrMOF nanoparticles before and after drug loading. (E) Temperature rise curves of different solutions under MW radiation (5 W, 5 min). (F) The maximum absorption peak of TMB with and without MW irradiation for Mn-ZrMOF.

declined post-MW radiation, indicating that Mn-ZrMOF nanoparticles can enhance the tumoricidal effects of MW (Fig. 3A).

Subsequently, we employed 2',7'-dichlorofluorescin diacetate (DCFH-DA) as a ROS probe to detect ROS in the treated MB49 cells. The results, depicted in Fig. 3B, revealed no ROS production in the untreated control group and the group exposed only to MW radiation. A trace amount of ROS was detected in the group with Mn-ZrMOF nanoparticles alone, while a substantial amount of ROS was found in the Mn-ZrMOF nanoparticles + MW radiation group. Studies have shown that the highly oxidative •OH, as a type of ROS, can exert toxic effects on cells when accumulating in large amounts, such as causing DNA breaks and protein inactivation [28]. Additionally, because hydrogen peroxide levels are significantly higher in the TME than in normal tissue, the catalytic

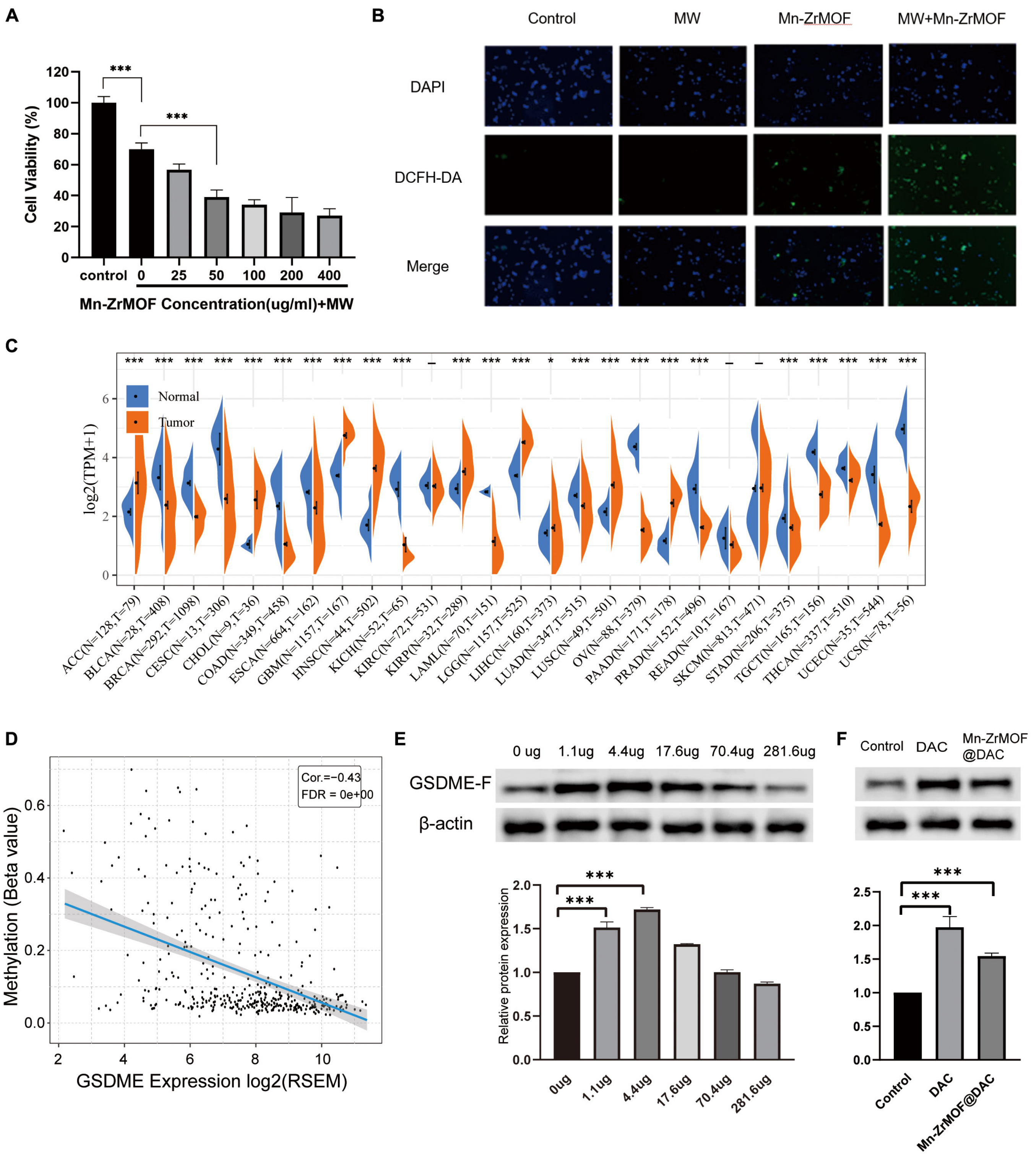

Fig. 3. Mn-ZrMOF@DAC inducing ROS production and up-regulating GSDME to achieve antitumor effects. (A) Inhibition of cell viability by different concentration gradients of Mn-ZrMOF nanoparticles under MW radiation (5 W, 5 min). (B) ROS staining results of different treatment groups, where green fluorescence signifies ROS production. (C) Expression of GSDME in various tumors, with low expression of GSDME in tumor tissues of bladder cancer patients. (D) Negative correlation between GSDME expression and methylation level in bladder cancer. (E) Protein expression of GSDME after treatment with different concentrations of DAC in MB49 cells, and (F) protein expression following treatment with DAC-loaded Mn-ZrMOF@DAC.

reaction uses the high concentration of hydrogen peroxide within tumor cells as a substrate, and local microwave radiation enhances the catalytic efficiency [29]. We have preliminarily proven that the Mn-ZrMOF nanoparticles synthesized in this study can enhance the effectiveness of microwave therapy by boosting the MW thermal effect and inducing ROS production under MW radiation. Possessing good biocompatibility, these nanoparticles can serve as sensitizers for microwave therapy.

Design of Mn-ZrMOF@DAC material to up-regulate expression of the pyroptosis key gene GSDME

Regrettably, the cellular damage and necrosis caused by microwave therapy have only acted as weak immunostimulatory factors against tumor immunity. Pyroptosis, as a newly discovered form of immunogenic cell death, is capable of promoting antitumor immunity [17]. However, most tumor cells are restricted from activating pyroptosis due to low expression of the key protein GSDME in the pyroptosis pathway [23]. Indeed, utilizing the TCGA database, we validated the expression of GSDME across various cancers and found GSDME to be significantly down-regulated in bladder, breast, cervical, colon, renal clear cell, ovarian, prostate, endometrial, and uterine sarcoma cancers (Fig. 3C). Previous research indicates that GSDME expression is negatively regulated by DNA methylation [23]. In bladder cancer, we indeed found a negative correlation between the expression of GSDME and the level of gene methylation (Fig. 3D). Therefore, this study was designed to use the DNMT inhibitor DAC to up-regulate the expression of GSDME in tumor cells, thereby enhancing the pyroptotic activity in bladder cancer cells. We first assessed the toxic effects of DAC using a CCK8 assay, which showed that low concentrations of DAC had no toxic effect on cells (Fig. S3B). To determine the optimal concentration of DAC for use in MB49 cells, we used Western blot to detect the expression of GSDME in MB49 cells treated with different concentrations of DAC, finding that low concentrations of DAC (0.5 μM and 2.0 μM) significantly up-regulated GSDME expression. Consequently, we designed DAC-loaded Mn-ZrMOF@DAC nanoparticles and verified their ability to up-regulate GSDME expression in MB49 cells (Fig. 3E).

Mn-ZrMOF@DAC enhances the efficacy of MW therapy and induces pyroptosis

Having already proven that Mn-ZrMOF can synergistically enhance the antitumor effects of MW and that Mn-ZrMOF@DAC can induce the up-regulation of GSDM expression in bladder cancer, we will further explore whether Mn-ZrMOF@DAC can synergize with the antitumor effects of MW and induce pyroptosis in BLCA.

Initially, we divided MB49 cells into control, DAC, Mn-ZrMOF, MW, Mn-ZrMOF+MW, and Mn-ZrMOF@DAC+MW groups. These were incubated with normal medium, DAC, Mn-ZrMOF, and Mn-ZrMOF@DAC for 12 h, followed by MW treatment (5 W, 5 min) for the 3 groups requiring MW exposure. Based on drug loading rate and drug release curve, the concentration of DAC, Mn-ZrMOF, and Mn-ZrMOF@DAC in vitro experimentation was 3.3 μg/ml, 50 μg/ml, and 58.4 μg/ml. Live/dead cell staining results showed that the isolated low-concentration DAC group, Mn-ZrMOF group, and control group did not exhibit red fluorescent dead cells. The MW group presented a certain proportion of dead cells, whereas the Mn-ZrMOF+MW and Mn-ZrMOF@DAC+MW groups exhibited a noticeably higher proportion of dead cells compared to the MW group alone (Fig. 4A), indicating that Mn-ZrMOF+MW and Mn-ZrMOF@DAC+MW treatments have a stronger killing effect on MB49 cells. The CCK8 cell viability assay corroborated this result (Fig. 4C). In addition, we examined the toxic effects of Mn-ZrMOF@DAC and MW on normal bladder cells. Unfortunately, the results showed that there were still some toxic effects, but they were less severe than those of bladder cancer cells. We re-evaluated the toxic effect of DAC following microwave irradiation and found no significant difference (Fig. S3C). Therefore, we hope to utilize the enhanced permeability and retention effect of nanoparticles in solid tumors, the characteristics of high H_2O_2 in tumor tissues, and the regulation of accurate positioning and appropriate power of microwave therapy to achieve effective killing of tumor tissues while avoiding excessive damage to normal tissues.

By comparing cell morphological changes under the microscope between the Mn-ZrMOF+MW and Mn-ZrMOF@DAC+MW groups, we observed more pyroptotic cells with distinctive morphology, presenting as bubble-like swelling, in the Mn-ZrMOF@DAC+MW group (Fig. 4B).

In the preliminary phase of this study, Mn-ZrMOF@DAC was confirmed to up-regulate the expression of the key protein GSDME in the pyroptosis pathway. We further verified whether Mn-ZrMOF@DAC+MW treatment could activate GSDME-mediated pyroptosis. Western blot experiments revealed significant increases in GSDME in the DAC and Mn-ZrMOF@DAC groups, but activated GSDME-N was not detected. Activated GSDME-N and activated caspase-3 were only detected in the Mn-ZrMOF+MW and Mn-ZrMOF@DAC+MW groups, with visibly higher levels of activated GSDME-N in the Mn-ZrMOF@DAC+MW group (Fig. 4D). The identical outcomes were achieved in the tissue WB during animal experiments (Fig. S3D). In conjunction with previously verified mechanisms of Mn-ZrMOF+MW inducing ROS, this suggests that during Mn-ZrMOF@DAC+MW treatment, DAC loaded in Mn-ZrMOF@DAC can be released into tumor cells, up-regulating GSDME protein, and massive ROS production post-MW radiation can activate caspase-3. The activated caspase-3 cleaves GSDME to form the pore-forming active GSDME-N, which perforates the tumor cell membrane, ultimately leading to tumor cell pyroptosis. Simultaneously, we detected the release of cellular contents LDH and ATP in the supernatants of cultured cells from each group. As shown in Fig. 4E and F, the release of LDH and ATP was significantly higher after Mn-ZrMOF@DAC+MW treatment compared to other groups. Simultaneously, we observed consistent results in experiments involving human bladder cancer cell UMUC-3 (Fig. S4). We found that the Mn-ZrMOF@DAC+MW group exhibited an increase in vesicular swelling cells, which is associated with pyroptosis (Fig. S4A). We tested the cell viability after treatment in each group, as well as the release of cellular contents lactate dehydrogenase (LDH) and adenosine triphosphate (ATP) in the cell supernatant. Compared with other groups, Mn-ZrMOF@DAC+MW treatment significantly reduced cell viability and significantly increased the release of LDH and ATP (Figure S4B, D, E). Activation of GSDME-N and caspase-3 was observed exclusively in the Mn-ZrMOF+MW and Mn-ZrMOF@DAC+MW groups, with higher expression levels of activated GSDME-N noted in the Mn-ZrMOF@DAC+MW group (Figure S4C).

It is noteworthy that Mn-ZrMOF@DAC+MW did not result in increased ATP and LDH levels following the use of GSDME small-molecule inhibitors, indicating its inability to induce pyroptotic cell death. This underscores the significance of DAC-mediated up-regulation of GSDME as a pivotal step in this therapeutic approach (Fig. S5). After adding the GSDME small-molecule inhibitor SP600125, the results showed that the small molecule inhibitor significantly reduced the expression of GSDME (Figure S5G). The cell viability of each group after treatment was detected, as well as the release of LDH and ATP from the cell supernatant (Fig. S5A, C, E).Compared with thecontrol group, the cell viability of anti-GSDME was significantly increased, and the release of intracellular contents such as (LDH) and (ATP) in the cell supernatant was significantly decreased (Figure S5B, D, F).

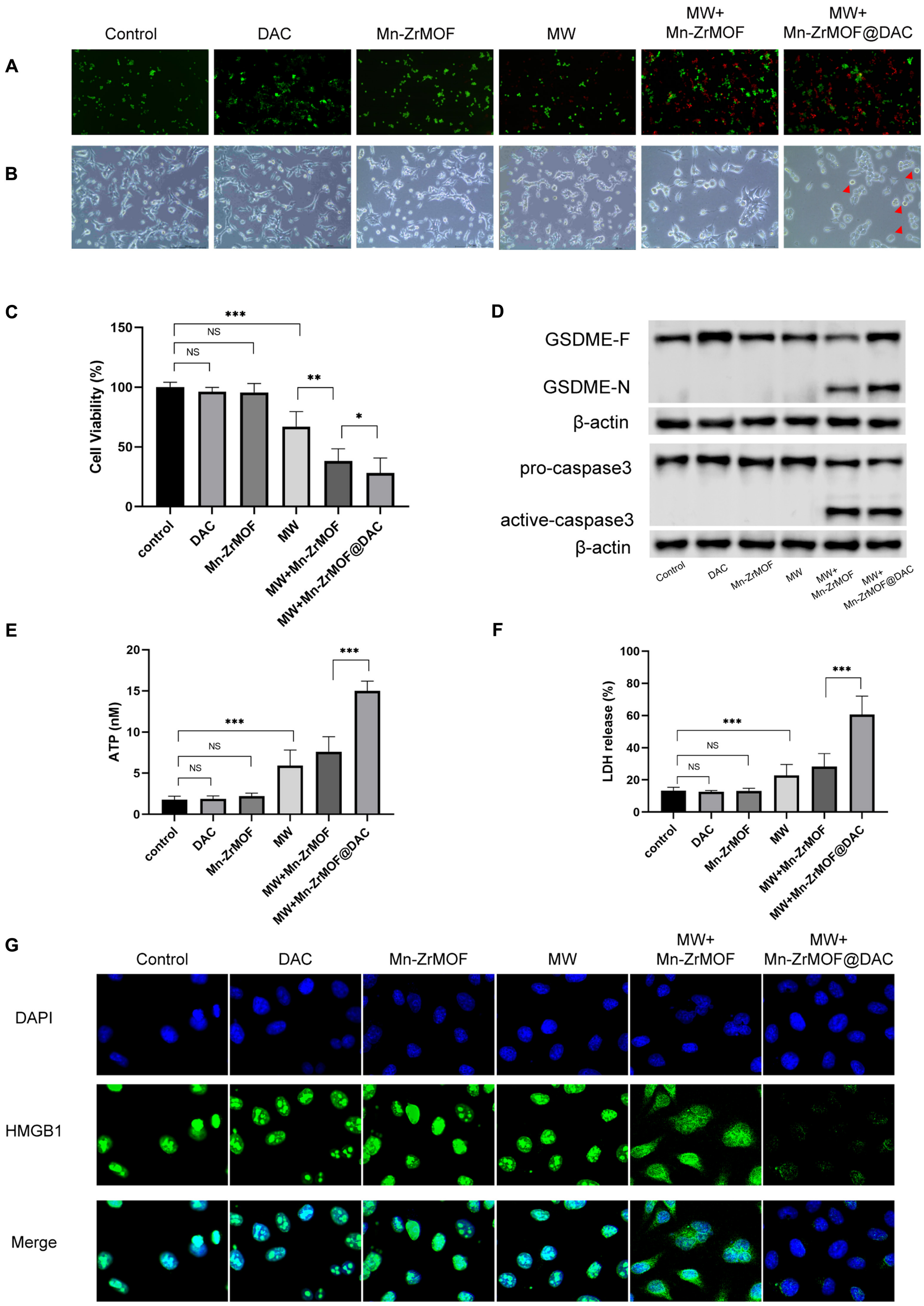

Fig. 4. Mn-ZrMOF@DAC inducing MB49 cell pyroptosis by up-regulating GSDME expression. (A) Live/dead staining results of cells from each group. (B) Cellular morphology under a light microscope for each group. (C) CCK8 assay results for cell viability in each group. (D) Western blot results for GSDME protein, caspase-3 protein, and their active fragments in each group. (E) ATP release assay results. (F) LDH release assay results. (G) Immunofluorescence results for HMGB1.

Additionally, immunofluorescence experiments revealed a marked reduction in the nuclear expression of High Mobility Group Box 1 (HMGB1) in the Mn-ZrMOF@DAC+MW group, suggesting the release of HMGB1 from the nucleus (Fig. 4G). As DAMPs, the release of HMGB1 and ATP can promote an immune response in the body [30,31]. Studies have shown that HMGB1 can lead to macrophage activation and tumor necrosis factor (TNF) secretion, which are involved in innate immune responses, and HMGB1 also participates in the migration of mature dendritic cells (DCs), which may be beneficial in triggering antitumor immune responses [32,33].

In vivo antitumor effects of microwave therapy based on Mn-ZrMOF@DAC in mice

This study successfully established a subcutaneous tumor animal model in C57 mice by subcutaneous injection of murine bladder cancer cells MB49, and the mice were randomly divided into 6 groups. When the subcutaneous tumor volume reached 100 mm^3, respective treatments were administered. For the groups requiring microwave radiation, mice were irradiated with microwaves (5 W, 5 min) 12 h after drug administration. Infrared thermography showed that the temperature in the tumor area of the Mn-ZrMOF@DAC+MW group rose faster and higher compared to the MW group alone (Fig. 5A and B). After treating the respective mouse groups, tumor volumes were recorded every other day to plot growth curves, and mice were euthanized after 12 days to compare and observe tumor masses. The model construction and treatment scheme are illustrated in Fig. 5C. The results shown in Fig. 5D and E indicate that there were no significant differences in tumor masses among the control, DAC, and Mn-ZrMOF groups, suggesting that DAC alone or Mn-ZrMOF nanoparticles alone do not exert direct antitumor effects. Compared to the MW group alone, the tumor ablation effect in the Mn-ZrMOF+MW group was significantly enhanced, and the tumor masses at euthanasia were noticeably smaller, indicating that Mn-ZrMOF can enhance the antitumor effects of MW. Notably, after MW radiation, elliptical necrosis formed around the subcutaneous tumors in both the Mn-ZrMOF+MW and Mn-ZrMOF@DAC+MW groups, the tumor volume decreased, and there was a significant difference in the tumor ablation effect

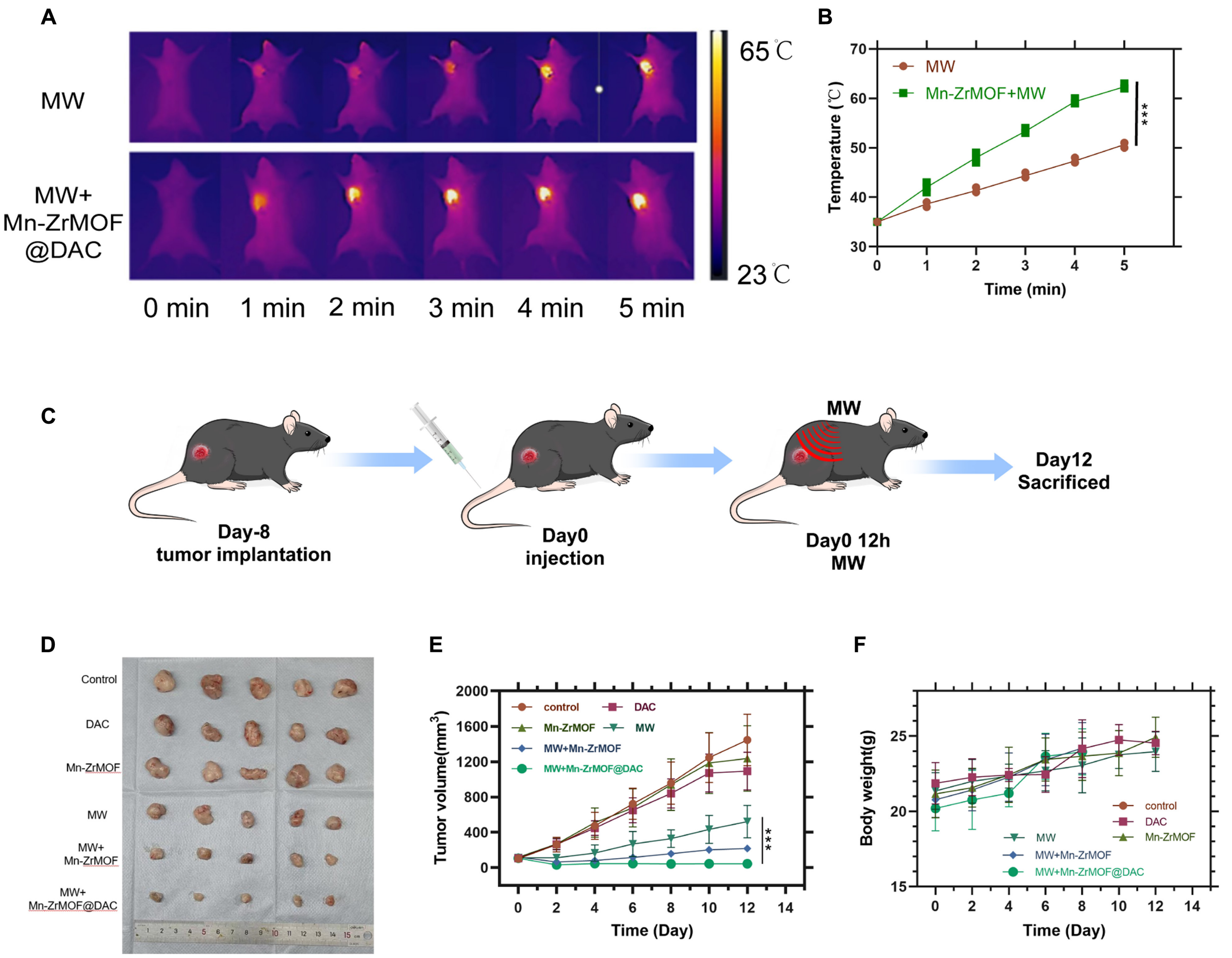

Fig. 5. Mn-ZrMOF@DAC enhances the in vivo antitumor effects of MW. (A) Infrared thermographic images of mice in each group under MW radiation (5 W, 5 min). (B) Temperature changes in the tumor sites of mice in each group under MW radiation (5 W, 5 min). (C) Images of tumor masses at euthanasia after 12 days of respective treatments in each group. (D) Changes in tumor volume in each group of mice. Five mice per group, $n = 5$. (E) Volume changes of primary tumors in each group after treatment. (F) Volume changes of distant tumors in each group after treatment; 5 mice per group, $n = 5$.

between the 2 groups. However, subsequent observations revealed that the growth of subcutaneous tumors in the Mn-ZrMOF@DAC+MW group was relatively slow, and ultimately, the tumor masses in the Mn-ZrMOF@DAC+MW group were significantly smaller than those in the other groups (Fig. 5D and E). These results suggest that Mn-ZrMOF@DAC+MW treatment not only has a significant tumor ablation effect but also inhibits tumor growth.

Antitumor immune response induced by microwave therapy based on Mn-ZrMOF@DAC in mice

To further investigate the in vivo antitumor immune response elicited by microwave therapy with Mn-ZrMOF@DAC, we quantitatively assessed the concentration of inflammatory factors in mouse serum using enzyme-linked immunosorbent assay. As depicted in Fig. 6A and B, the concentrations of IFN-γ and TNF-α in the Mn-ZrMOF@DAC+MW group were significantly higher than those in the control group, DAC group, Mn-ZrMOF group, MW group, and Mn-ZrMOF+MW group. Furthermore, after euthanizing the mice, a portion of tumor tissue was analyzed using flow cytometry to examine the changes in immune cell populations. The results showed a significant increase in the proportion of mature DCs (CD11c+CD80+CD86+) in the Mn-ZrMOF@DAC+MW group (Fig. 6C and F), which may be associated with the maturation of DCs triggered by the release of tumor antigens and inflammatory factors during pyroptosis, activating DCs and enhancing antigen presentation. Correspondingly, a significant increase in T lymphocytes (CD45+CD3+) within the mouse tumor tissue was detected (Fig. 6D and G), particularly the proportions of CD8+ T cells and CD4+ T cells (Fig. 6E, H, and I). Studies have shown that DCs are present within the TME, but there is an issue of impaired antigen cross-presentation, leading to immunological non-responsiveness. However, microwave therapy based on Mn-ZrMOF@DAC nanoparticles induces pyroptosis, releasing immunostimulatory factors and cytokines, such as type I interferons, TNFα, and interleukins, into the extracellular space [34]. The release of immunostimulatory factors and their recognition by pattern recognition receptors on antigen-presenting cells (such as DCs) lead to their transport to the cell surface of antigen-presenting cells, facilitating the transformation of DCs into mature DCs (CD80+CD86+). Activated DCs present tumor

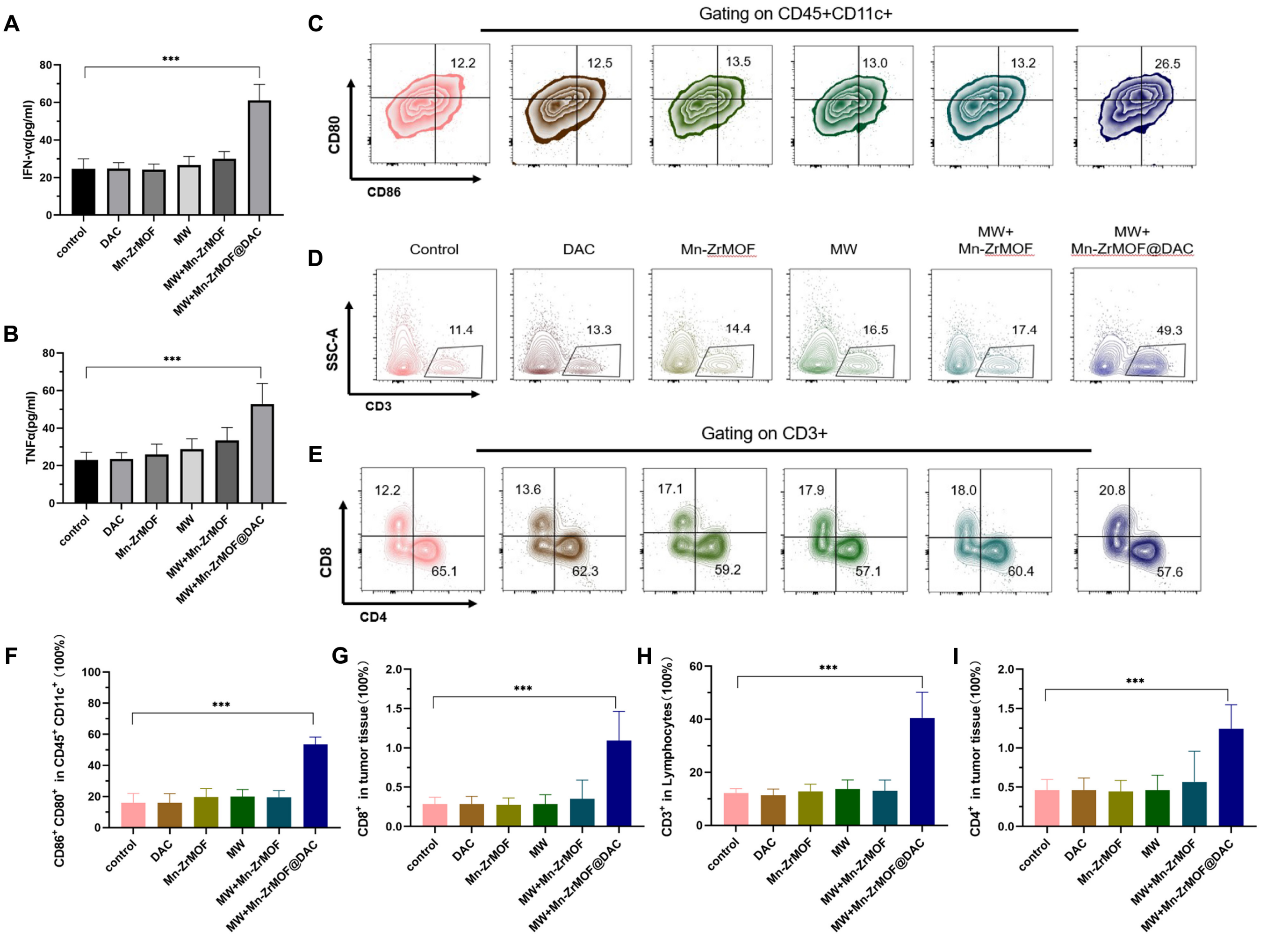

Fig. 6. Mn-ZrMOF@DAC enhances antitumor immune response during MW therapy. (A) Measurement of IFN-γ concentration. (B) Measurement of TNF-α concentration. (C) Flow cytometric scatter plot assessing mature DC cell infiltration in the tumor. (D) Scatter plot assessing CD3+ lymphocyte infiltration in the tumor. (E) Scatter plot assessing mature CD8+ T cell and CD4+ T cell infiltration in the tumor. (F) Proportion of mature DC cells (CD80+CD86+) within the total DC population (CD45+CD11c+) in tumor tissues of each group. (G) Proportion of CD3+T cells within the total cell count in tumor tissues of each group. (H) Proportion of CD3+CD8+T cells within the total cell count in tumor tissues of each group. (I) Proportion of CD3+CD4+T cells within the total cell count in tumor tissues of each group; 5 mice per group, $n = 5$.

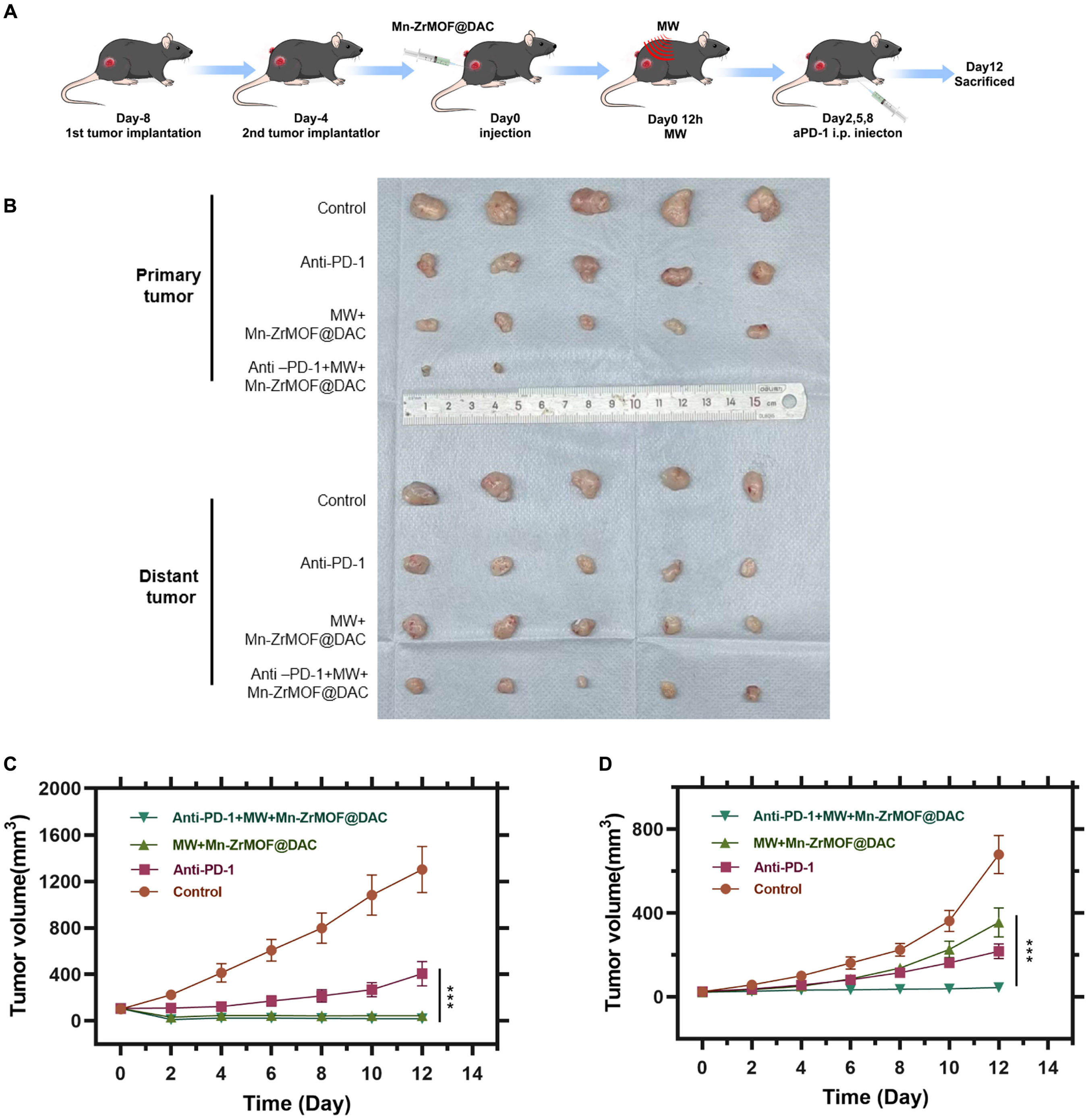

Fig. 7. Mn-ZrMOF@DAC enhances the efficacy of immunotherapy in microwave-immunotherapy synergistic treatment. (A) Schematic diagram of the bilateral tumor-bearing mouse model construction and treatment scheme. (B) Images of primary and distant tumors in each group of mice at day 12 post-treatment. (C) Volume changes of primary tumors in each group after treatment. (D) Volume changes of distant tumors in each group after treatment; 5 mice per group, $n = 5$.

antigens on MHC I or MHC II molecules to CD8+T cells and CD4+ T cells, respectively, and the body's antitumor immune effect is ultimately mediated by T lymphocytes (CD8+ cytotoxic T lymphocytes and CD4+ helper T cells) [35,36].

Microwave therapy based on Mn-ZrMOF@DAC can enhance the efficacy of anti-PD-1 immunotherapy

The infiltration of T cells in the TME is a key factor affecting the response to immune checkpoint inhibitor therapy. Given the above studies indicating that Mn-ZrMOF@DAC+MW treatment effectively promotes the recruitment of T cells, we established a bilateral tumor-bearing C57 mouse model of subcutaneous bladder cancer to further investigate its capacity to enhance the efficacy of immune checkpoint inhibitors. The model construction and treatment scheme are illustrated in Fig. 7A, where MB49 cells were injected subcutaneously on one side as the primary tumor, and 4 days later, MB49 cells were injected on the opposite side as the distant tumor. Group treatments were administered only to the primary tumor, with Mn-ZrMOF@DAC+MW intended as a pretreatment to improve αPD-1 therapy response. Thus,

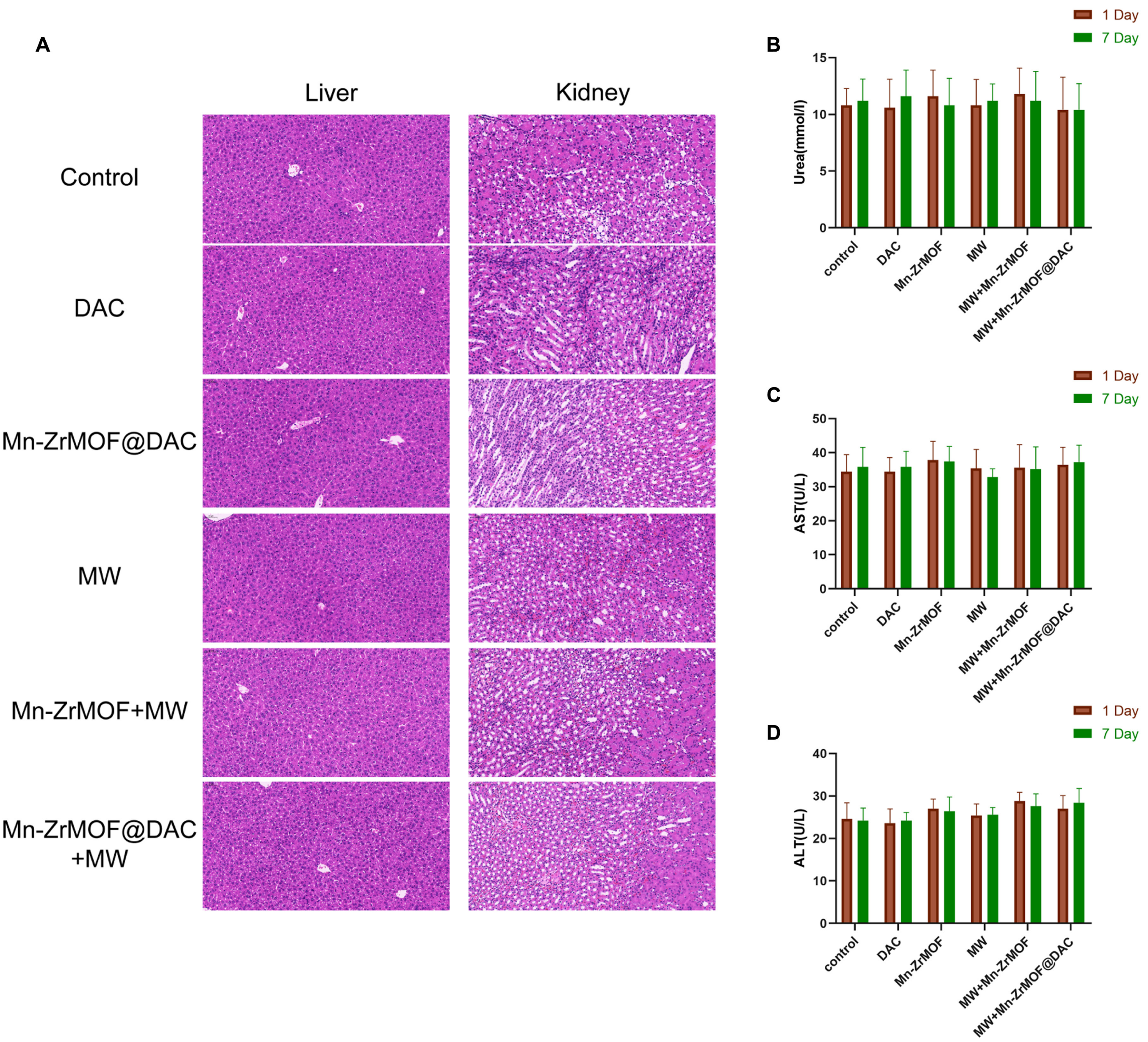

Fig. 8. In vivo safety of Mn-ZrMOF@DAC. (A) H&E staining of the liver and kidneys in each group of mice after treatment; 5 mice per group, $n = 5$. (B) Blood urea nitrogen levels in each group of mice after treatment. (C) AST levels in the blood of each group of mice after treatment. (D) ALT levels in the blood of each group of mice after treatment.

Mn-ZrMOF@DAC was first injected, followed by local MW irradiation (5 W, 5 min) of the primary tumor after 12 h, and αPD-1 was injected 1 day later. Tumor size was monitored daily, and we found that compared to the control group, the Mn-ZrMOF@DAC+MW group, αPD-1 group, and Mn-ZrMOF@DAC+MW+αPD-1 group all inhibited the growth of both the primary and distant tumors (Fig. 7B to D and Table S1). Notably, the combined treatment of Mn-ZrMOF@DAC+MW+αPD-1 was the most effective, with the primary tumors in 3 mice regressing after treatment, and the distant tumors in the Mn-ZrMOF@DAC+MW+αPD-1 group showing the greatest suppression. This indicates the remarkable success of the combined Mn-ZrMOF@DAC+MW+αPD-1 therapy. The growth of the distant tumor was significantly inhibited by injecting ZrMOF@DAC nanoparticles and performing microwave irradiation on only one side, suggesting that the microwave therapy based on Mn-ZrMOF@DAC nanoparticles induced an immunogenic cell death effect, generating a systemic antitumor immune response beneficial for suppressing tumor metastasis and recurrence. Additionally, we found that microwave therapy based on Mn-ZrMOF@DAC nanoparticles could significantly enhance the efficacy of anti-PD-1 immune checkpoint inhibitors, which is of great importance for advancing the development of new combined immunotherapeutic strategies.

Biosafety assessment

To verify the in vivo safety of Mn-ZrMOF@DAC, we monitored the body weight changes of the mice in each group, collected peripheral blood for liver and kidney function tests, and gathered major organs for histopathological examination after euthanizing the mice. All tests and examinations indicated that relative to the control group, there were no significant changes

in body weight, liver and kidney functions, or visceral toxicity (Fig. 8).

Discussion

Bladder cancer is one of the 10 most common cancers in the and surgery, the prognosis of patients with advanced bladder cancer is still poor [37]. At present, studies have confirmed that cancer immunotherapy, including immunocheckpoint blocking therapy [38], can achieve good survival benefits for patients with advanced bladder cancer, but only a few patients are sensitive to immunocheckpoint inhibitors [39]. In addition, in recent years, it has been found that cell pyroptosis releases tumor antigens, induces tumor immunity [40], and improves the efficacy of immune checkpoint inhibitors. Cellular pyroptosis is defined as programmed cell death mediated by the perforation protein GSDM family [41], characterized by DNA breakage and chromatin condensation, accompanied by cell swelling and membrane rupture. At present, research has found that the molecular mechanisms of cell pyroptosis mainly include the classical pathway, the non-classical pathway, the caspase-3/caspase-8-mediated alternative pathway, and the granzyme-mediated alternative pathway [42]. The GSDM protein family plays an executor role in various pathways of cell pyroptosis [43], but the exact mechanism and role of regulating the GSDM protein family still need further research.

In this study, a zirconium-based organic metal framework doped with manganese ions was prepared by the hydrothermal method. Mn-ZrMOF@DAC nanoparticles have good biocompatibility and can rapidly release DAC under acidic conditions. As a sensitizer for microwave therapy [44], they can effectively promote the thermal effect of microwave radiation. At the same time, microwaving enhanced the peroxidase catalytic activity of Mn-ZrMOF nanoparticles and generated a large amount of ROS to achieve the effect of killing tumor cells. The synthesized Mn-ZrMOF nanoparticles can enhance the role of microwave therapy in killing tumor cells by enhancing the thermal effect of MW and inducing ROS under MW, and this is the innovative application of Mn-ZrMOF@DAC nanoparticles in the treatment of bladder cancer, while the loaded DAC can cause GSDME-mediated proinflammatory cell death of tumor cells. In view of the fact that anti-PDL1/PD-1 treatment can significantly improve the survival and prognosis of bladder cancer patients, the findings of this study are based on Mn-ZrMOF@DAC. Microwave therapy with nanoparticles can enhance the efficacy of anti-PD-1 immune checkpoint inhibitors [45], generate systemic immune effects, and inhibit tumor metastasis and recurrence. This study has developed a new treatment strategy for bladder cancer that not only has a significant ablation effect on bladder cancer but also can activate antitumor immunity and enhance the efficacy of immunotherapy, providing new ideas for patients with bladder cancer, especially those who are not sensitive to immunotherapy.

To emphasize the points we have raised about the specificity of Mn-ZrMOF@DAC nanoparticles, we compared our work with other studies in this field, such as Fu et al. [14], which demonstrated that Mn-ZrMOF@DAC can achieve efficient treatment of liver cancer cells HepG2 through the synergistic effect of thermal therapy and dynamic therapy. Compared to our research, these works explore different materials and assembly methods research, and unlike their methods for different types of cancer, our work provides new ideas for enhancing the immune efficacy of bladder cancer. In addition, Mn-ZrMOF nanomaterials were first used in the study of bladder cancer. Through the microwave treatment of nanoparticles, we induced the pyroptosis of bladder cancer cell MB49 and enhanced the efficacy of anti-PD-1 immune checkpoint inhibitor. This innovative method enables us to achieve a strong antitumor effect. In general, our research shows the originality of developing a nano platform by assembling nanoparticles, which are specially tailored for the treatment of bladder cancer. Nanomedicine plays an important role in the diagnosis and treatment of cancer, utilizing the targeting and sustained-release properties of nanoparticles to improve drug efficacy and safety, while reducing side effects and toxicity [46].

Acknowledgments

The authors thank M.J. for her help with polishing the article.
Funding: This work was supported by the National Natural Science Foundation of China (81902592, 82070785, 82303760, and 82373337), the China Postdoctoral Innovation Talents Support Program (BX20230431), the China Postdoctoral Science Foundation (2023M733951), the Hunan Natural Science Foundation (2023JJ30877, 2023JJ40946, and 2024JJ2093), the Hunan Province Young Talents Program (2021RC3027 and 2023RC3073), the Changsha Natural Science Foundation (kq2208377), and the Youth Science Foundation of Xiangya Hospital (2022Q20).
Author contributions: The article's conception and design were created by J. Hu, J.C., and X.Z. The investigations were carried out by N.G., Z.L., and Z.Y. H.D. and C.Z. were mainly accountable for both the data analysis and article analysis. J. Huang and X.G. provided experimental materials for this project. M.J. and J.X. revised the manuscript. All authors read and approved the final manuscript.
Competing interests: The authors declare that they have no competing interests.

Data Availability

The data reported in the manuscript and Supplementary Material is available upon request from Pei Yuin Keng at keng.py@gapp.nthu.edu.tw.

Supplementary Materials

Figs. S1 to S5
Table S1

References

1. Lenis AT, Lec PM, Chamie K, Mshs MD. Bladder cancer: A review. *JAMA*. 2020;324(19):1980–1991.
2. D'Andrea D, Black PC, Zargar H, Dinney CP, Soria F, Cookson MS, Montgomery JS, Kassouf W, Dall'Era MA, Sridhar SS, et al. Identifying the optimal number of neoadjuvant chemotherapy cycles in patients with muscle invasive bladder cancer. *J Urol*. 2022;207(1):70–76.
3. Powles T, Kockx M, Rodriguez-Vida A, Duran I, Crabb SJ, Van Der Heijden MS, Szabados B, Pous AF, Gravis G, Herranz UA, et al. Clinical efficacy and biomarker analysis of neoadjuvant atezolizumab in operable urothelial carcinoma in the ABACUS trial. *Nat Med*. 2019;25(11):1706–1714.

4. Song D, Powles T, Shi L, Zhang L, Ingersoll MA, Lu YJ. Bladder cancer, a unique model to understand cancer immunity and develop immunotherapy approaches. *J Pathol*. 2019;249(2):151–165.
5. Cathomas R, Lorch A, Bruins HM, Compérat EM, Cowan NC, Efstathiou JA, Fietkau R, Gakis G, Hernández V, Espinós EL, et al. The 2021 updated European Association of Urology guidelines on metastatic urothelial carcinoma. *Eur Urol*. 2022;81(1):95–103.
6. Yu WD, Sun G, Li J, Xu J, Wang X. Mechanisms and therapeutic potentials of cancer immunotherapy in combination with radiotherapy and/or chemotherapy. *Cancer Lett*. 2019;452:66–70.
7. Shu G, Wang H, Zhao HX, Zhang X. Microwave-assisted synthesis of black titanium monoxide for synergistic tumor phototherapy. *ACS Appl Mater Interfaces*. 2019;11(3):3323–3333.
8. Yao A, Jiao X, Chen D, Li C. Photothermally enhanced detoxification of chemical warfare agent simulants using bioinspired core-shell dopamine-melanin@metal-organic frameworks and their fabrics. *ACS Appl Mater Interfaces*. 2019;11(8):7927–7935.
9. Chen X, Tan L, Liu T, Meng X. Micro-nanomaterials for tumor microwave hyperthermia: Design, preparation, and application. *Curr Drug Deliv*. 2017;14(3):307–322.
10. Jin L, Liu X, Zheng Y, Zhang Y, Li Z, Zhu S, Jiang H, Cui Z, Wu S. Interfacial and defect polarization enhanced microwave noninvasive therapy for *Staphylococcus aureus*-infected chronic osteomyelitis. *ACS Nano*. 2023;17(18):18200–18216.
11. Jin L, Zheng Y, Liu X, Zhang Y, Li Z, Liang Y, Zhu S, Jiang H, Cui Z, Wu S. Magnetic composite rapidly treats *Staphylococcus aureus*-infected osteomyelitis through microwave strengthened thermal effects and reactive oxygen species. *Small*. 2022;18(41):e2204028.
12. Wu Q, Li T, Song J, Sun X, Ren X, Fu C, Chen L, Tan L, Niu M, Meng X. A novel instantaneous self-assembled hollow MOF-derived nanodrug for microwave thermo-chemotherapy in triple-negative breast cancer. *ACS Appl Mater Interfaces*. 2022;14(46):51656–51668.
13. Zhu Y, Yang Z, Pan Z, Hao Y, Wang C, Dong Z, Li Q, Han Y, Tian L, Feng L, et al. Metallo-alginate hydrogel can potentiate microwave tumor ablation for synergistic cancer treatment. *Sci Adv*. 2022;8(31):eabo5285.
14. Fu C, Zhou H, Tan L, Huang Z, Wu Q, Ren X, Ren J, Meng X. Microwave-activated Mn-doped zirconium metal-organic framework nanocubes for highly effective combination of microwave dynamic and thermal therapies against cancer. *ACS Nano*. 2018;12(3):2201–2210.
15. Slovak R, Ludwig JM, Gettinger SN, Herbst RS, Kim HS. Immuno-thermal ablations—Boosting the anticancer immune response. *J Immunother Cancer*. 2017;5(1):78.
16. Shi J, Gao W, Shao F. Pyroptosis: Gasdermin-mediated programmed necrotic cell death. *Trends Biochem Sci*. 2017;42(4):245–254.
17. Gao W, Wang X, Zhou Y, Wang X, Yu Y. Autophagy, ferroptosis, pyroptosis, and necroptosis in tumor immunotherapy. *Signal Transduct Target Ther*. 2022;7(1):196.
18. Wang Y, Gao W, Shi X, Ding J, Liu W, He H, Wang K, Shao F. Chemotherapy drugs induce pyroptosis through caspase-3 cleavage of a gasdermin. *Nature*. 2017;547(7661):99–103.
19. Xiao Y, Zhang T, Ma X, Yang QC, Yang LL, Yang SC, Liang M, Xu Z, Sun ZJ. Microenvironment-responsive prodrug-induced pyroptosis boosts cancer immunotherapy. *Adv Sci (Weinh)*. 2021;8(24):e2101840.
20. Wang H, Rong X, Zhao G, Zhou Y, Xiao Y, Ma D, Jin X, Wu Y, Yan Y, Yang H, et al. The microbial metabolite trimethylamine N-oxide promotes antitumor immunity in triple-negative breast cancer. *Cell Metab*. 2022;34(4):581–594.e588.
21. Zhang S, Zhang Y, Feng Y, Wu J, Hu Y, Lin L, Xu C, Chen J, Tang Z, Tian H, et al. Biomineralized two-enzyme nanoparticles regulate tumor glycometabolism inducing tumor cell pyroptosis and robust antitumor immunotherapy. *Adv Mater*. 2022;34(50):e2206851.
22. Rogers C, Fernandes-Alnemri T, Mayes L, Alnemri D, Cingolani G, Alnemri ES. Cleavage of DFNA5 by caspase-3 during apoptosis mediates progression to secondary necrotic/pyroptotic cell death. *Nat Commun*. 2017;8:14128.
23. Fan JX, Deng RH, Wang H, Liu XH, Wang XN, Qin R, Jin X, Lei TR, Zheng D, Zhou PH, et al. Epigenetics-based tumor cells pyroptosis for enhancing the immunological effect of chemotherapeutic nanocarriers. *Nano Lett*. 2019;19(11):8049–8058.
24. Akino K, Toyota M, Suzuki H, Imai T, Maruyama R, Kusano M, Nishikawa N, Watanabe Y, Sasaki Y, Abe T, et al. Identification of DFNA5 as a target of epigenetic inactivation in gastric cancer. *Cancer Sci*. 2007;98(1):88–95.
25. Patnaik E, Madu C, Lu Y. Epigenetic modulators as therapeutic agents in cancer. *Int J Mol Sci*. 2023;24(19):14964.
26. Li X, Zhang Y, Chen M, Mei Q, Liu Y, Feng K, Jia H, Dong L, Shi L, Liu L, et al. Increased IFNγ(+) T cells are responsible for the clinical responses of low-dose DNA-demethylating agent decitabine antitumor therapy. *Clin Cancer Res*. 2017;23(20):6031–6043.
27. Lubner MG, Brace CL, Hinshaw JL, Lee FT Jr. Microwave tumor ablation: Mechanism of action, clinical results, and devices. *J Vasc Interv Radiol*. 2010;21(8 Suppl):S192–S203.
28. Zhou R, Yazdi AS, Menu P, Tschopp J. A role for mitochondria in NLRP3 inflammasome activation. *Nature*. 2011;469(7329):221–225.
29. Tang Z, Liu Y, He M, Bu W. Chemodynamic therapy: Tumour microenvironment-mediated Fenton and Fenton-like reactions. *Angew Chem Int Ed Engl*. 2019;58(4):946–956.
30. Apetoh L, Ghiringhelli F, Tesniere A, Criollo A, Ortiz C, Lidereau R, Mariette C, Chaput N, Mira JP, Delaloge S, et al. The interaction between HMGB1 and TLR4 dictates the outcome of anticancer chemotherapy and radiotherapy. *Immunol Rev*. 2007;220:47–59.
31. Ma Y, Adjemian S, Mattarollo SR, Yamazaki T, Aymeric L, Yang H, Portela Catani JP, Hannani D, Duret H, Steegh K, et al. Anticancer chemotherapy-induced intratumoral recruitment and differentiation of antigen-presenting cells. *Immunity*. 2013;38(4):729–741.
32. Li H, Li J, Zhang G, Da Q, Chen L, Yu S, Zhou Q, Weng Z, Xin Z, Shi L, et al. HMGB1-induced p62 overexpression promotes snail-mediated epithelial-mesenchymal transition in glioblastoma cells via the degradation of GSK-3β. *Theranostics*. 2019;9(7):1909–1922.
33. Deng H, Yang W, Zhou Z, Tian R, Lin L, Ma Y, Song J, Chen X. Targeted scavenging of extracellular ROS relieves suppressive immunogenic cell death. *Nat Commun*. 2020;11(1):4951.
34. Feng S, Fox D, Man SM. Mechanisms of gasdermin family members in inflammasome signaling and cell death. *J Mol Biol*. 2018;430(18 Pt B):3068–3080.

35. Yu M, Wang H, Ding A, Golenbock DT, Latz E, Czura CJ, Fenton MJ, Tracey KJ, Yang H. HMGB1 signals through toll-like receptor (TLR) 4 and TLR2. *Shock*. 2006;26(2):174–179.
36. Zhang L, Yang QC, Wang S, Xiao Y, Wan SC, Deng H, Sun ZJ. Engineering multienzyme-mimicking covalent organic frameworks as pyroptosis inducers for boosting antitumor immunity. *Adv Mater*. 2022;34(13):e2108174.
37. Dyrskjøt L, Hansel DE, Efstathiou JA, Knowles MA, Galsky MD, Teoh J, Theodorescu D. Bladder cancer. *Nat Rev Dis Primers*. 2023;9(1):58.
38. Lopez-Beltran A, Cookson MS, Guercio BJ, Cheng L. Advances in diagnosis and treatment of bladder cancer. *BMJ*. 2024;384:e076743.
39. Robertson AG, Meghani K, Cooley LF, McLaughlin KA, Fall LA, Yu Y, Castro MAA, Groeneveld CS, de Reyniès A, Nazarov VI, et al. Expression-based subtypes define pathologic response to neoadjuvant immune-checkpoint inhibitors in muscle-invasive bladder cancer. *Nat Commun*. 2023;14(1):2126.
40. Wei X, Xie F, Zhou X, Wu Y, Yan H, Liu T, Huang J, Wang F, Zhou F, Zhang L. Role of pyroptosis in inflammation and cancer. *Cell Mol Immunol*. 2022;19(9):971–992.
41. Ren Y, Feng M, Hao X, Liu X, Li J, Li P, Gao J, Qi Q, Du L, Wang C, et al. USP48 stabilizes gasdermin E to promote pyroptosis in cancer. *Cancer Res*. 2023;83(7):1074–1093.
42. Rao Z, Zhu Y, Yang P, Chen Z, Xia Y, Qiao C, Liu W, Deng H, Li J, Ning P, et al. Pyroptosis in inflammatory diseases and cancer. *Theranostics*. 2022;12(9):4310–4329.
43. Aglietti RA, Dueber EC. Recent insights into the molecular mechanisms underlying pyroptosis and gasdermin family functions. *Trends Immunol*. 2017;38(4):261–271.
44. Cui R, Wang L, Zhang D, Zhang K, Dou J, Dong L, Zhang Y, Wu J, Tan L, Yu J, et al. Combination therapy using microwave ablation and d-mannose-chelated iron oxide nanoparticles inhibits hepatocellular carcinoma progression. *Acta Pharm Sin B*. 2022;12(9):3475–3485.
45. Salomé B, Sfakianos JP, Ranti D, Daza J, Bieber C, Charap A, Hammer C, Banchereau R, Farkas AM, Ruan DF, et al. NKG2A and HLA-E define an alternative immune checkpoint axis in bladder cancer. *Cancer Cell*. 2022;40(9):1027–1043.e1029.
46. Cheng Z, Li M, Dey R, Chen Y. Nanomaterials for cancer therapy: Current progress and perspectives. *J Hematol Oncol*. 2021;14(1):85.
