# Supplementary figures and images for: Nanotherapeutic System with Effective Microwave Sensitization and Pyroptosis Programming Enable Synergistic Microwave-Immunotherapy in Bladder Cancer
Source: Biomater Res. 2024 Sep 10;28:0077. doi: 10.34133/bmr.0077 (PMC12237075; doi:10.34133/bmr.0077)

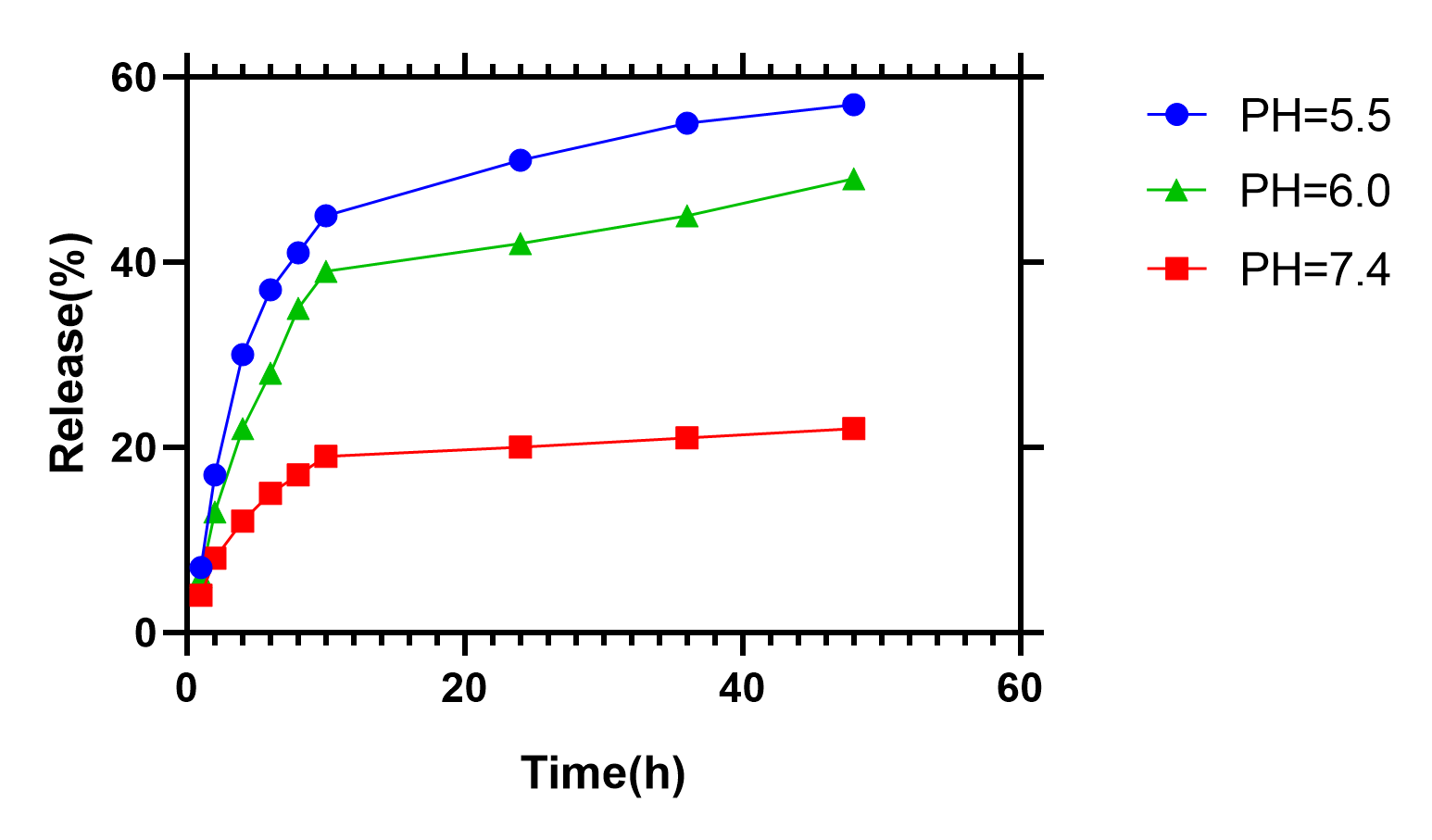

Supplement: Supplementary 1 — Figs. S1 to S5 Table S1 [file bmr.0077.f1.zip › S1.tif]

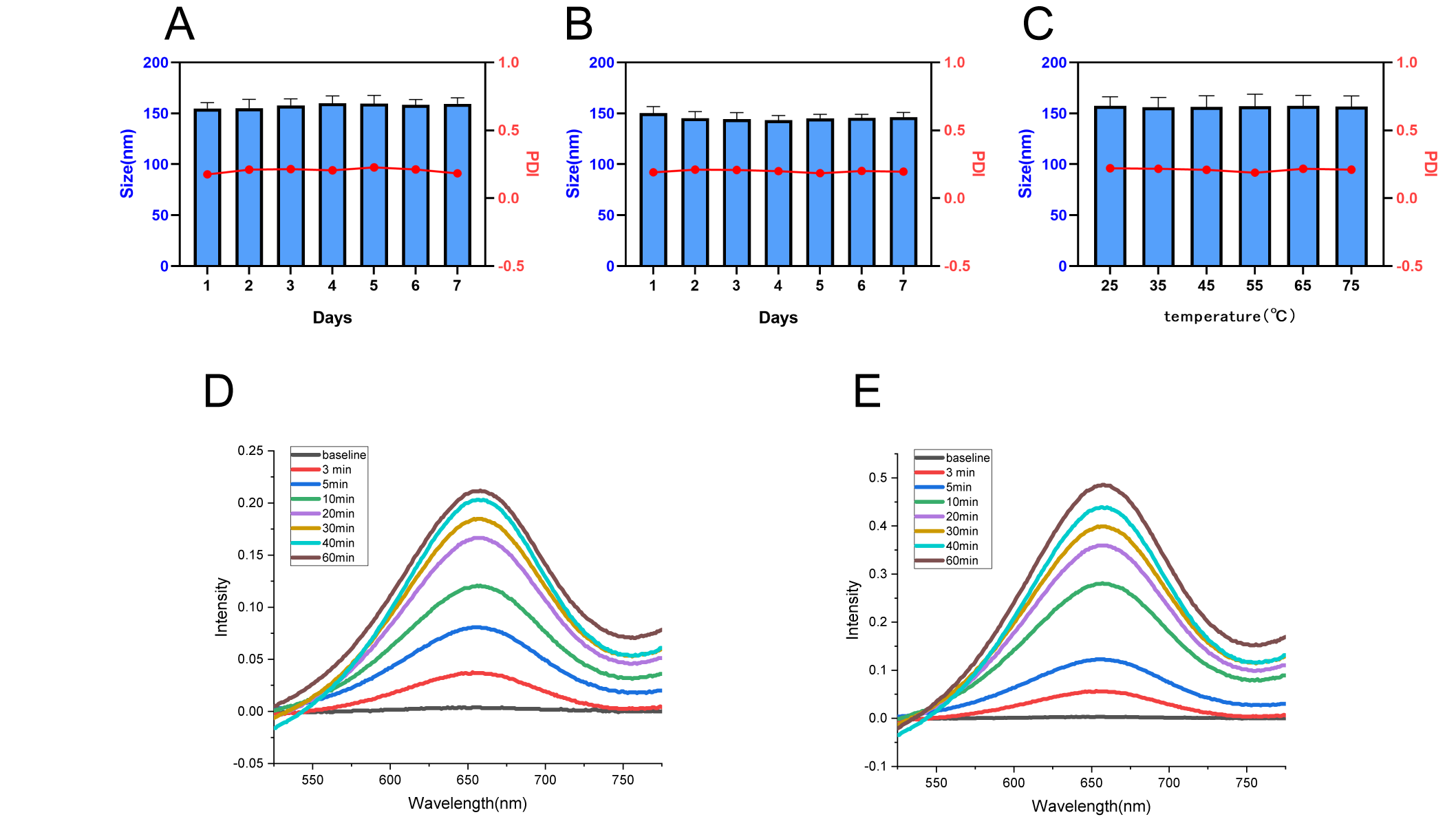

Supplement: Supplementary 1 — Figs. S1 to S5 Table S1 [file bmr.0077.f1.zip › S2.tif]

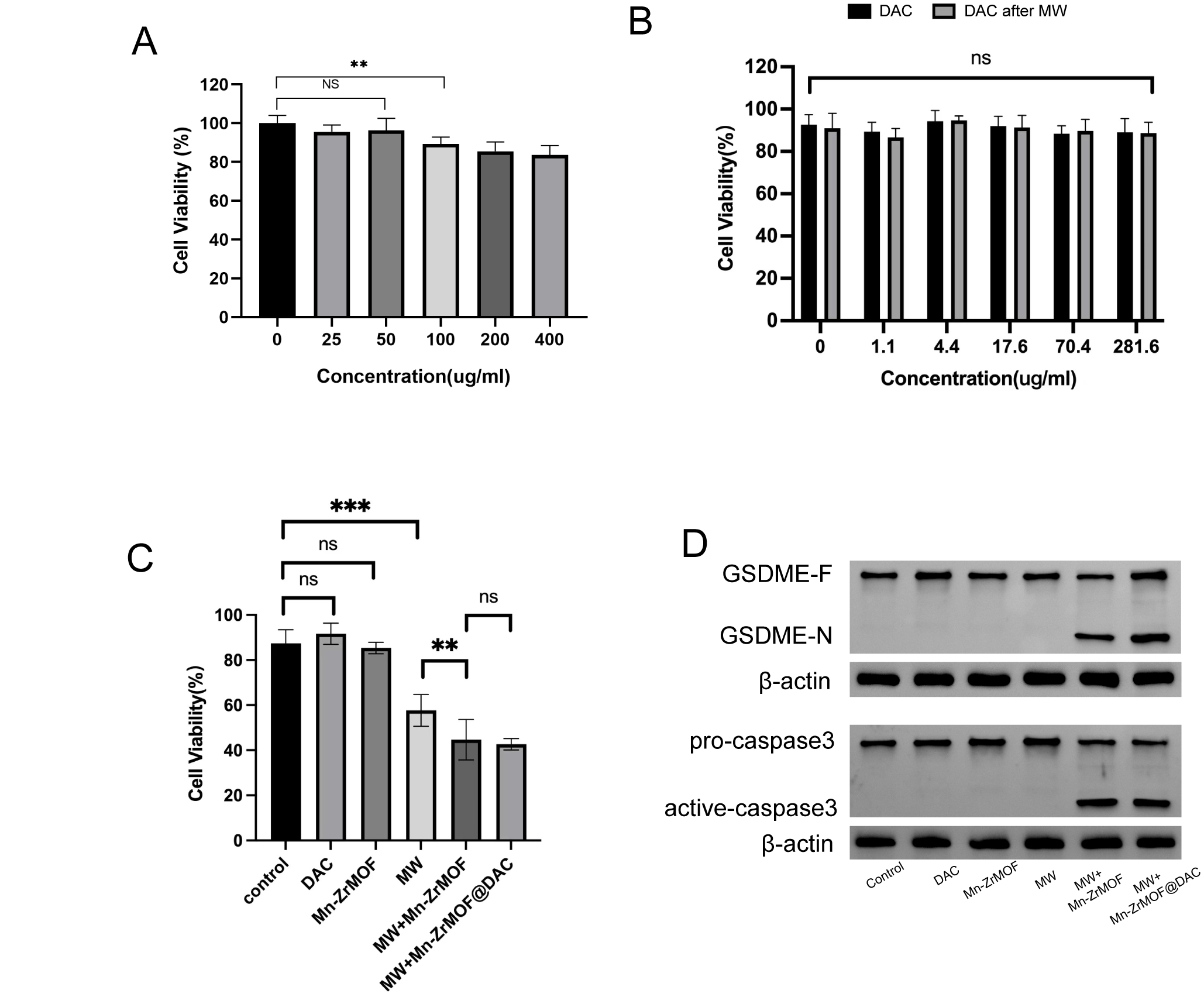

Supplement: Supplementary 1 — Figs. S1 to S5 Table S1 [file bmr.0077.f1.zip › S3.tif]

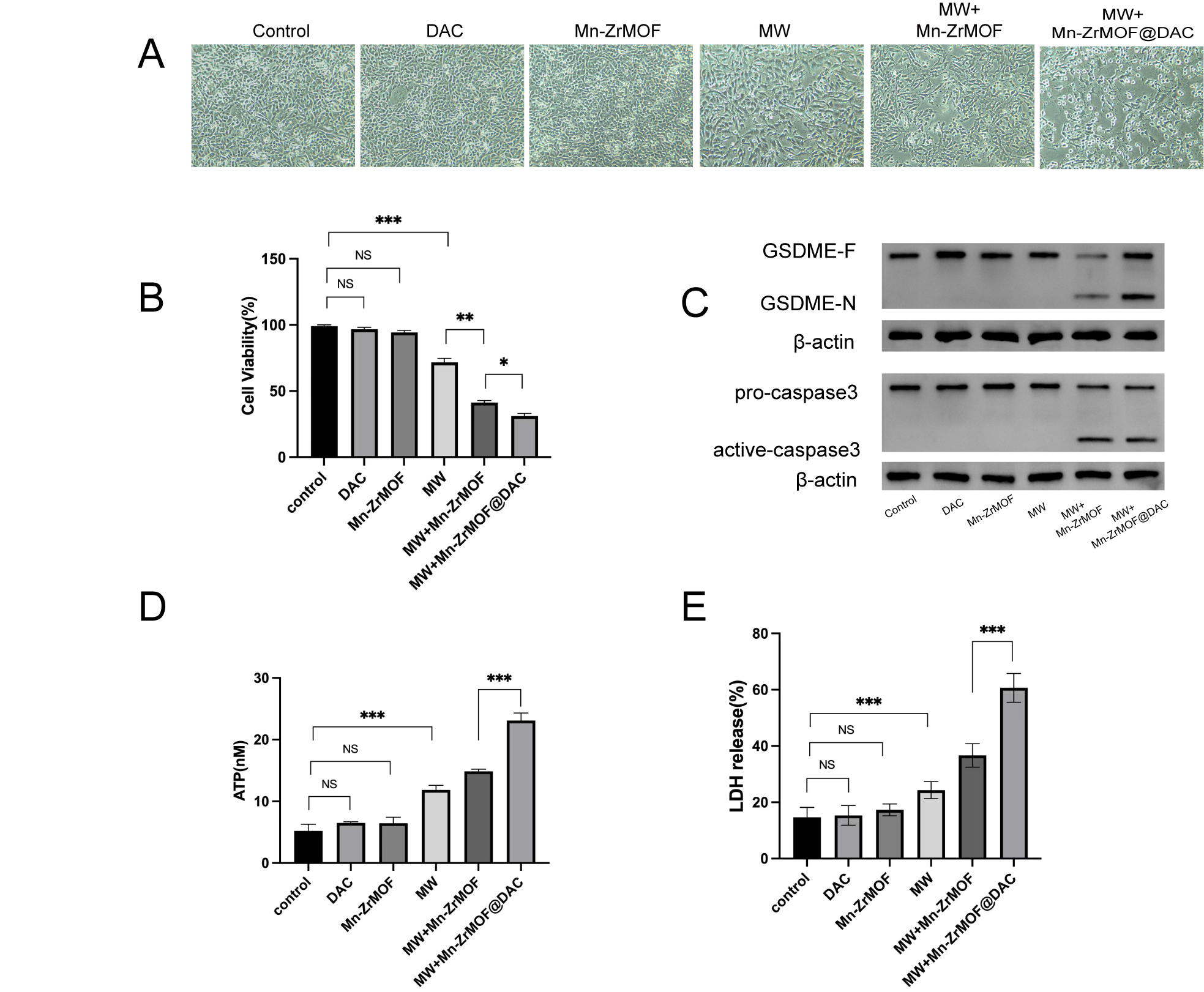

Supplement: Supplementary 1 — Figs. S1 to S5 Table S1 [file bmr.0077.f1.zip › S4.tif]

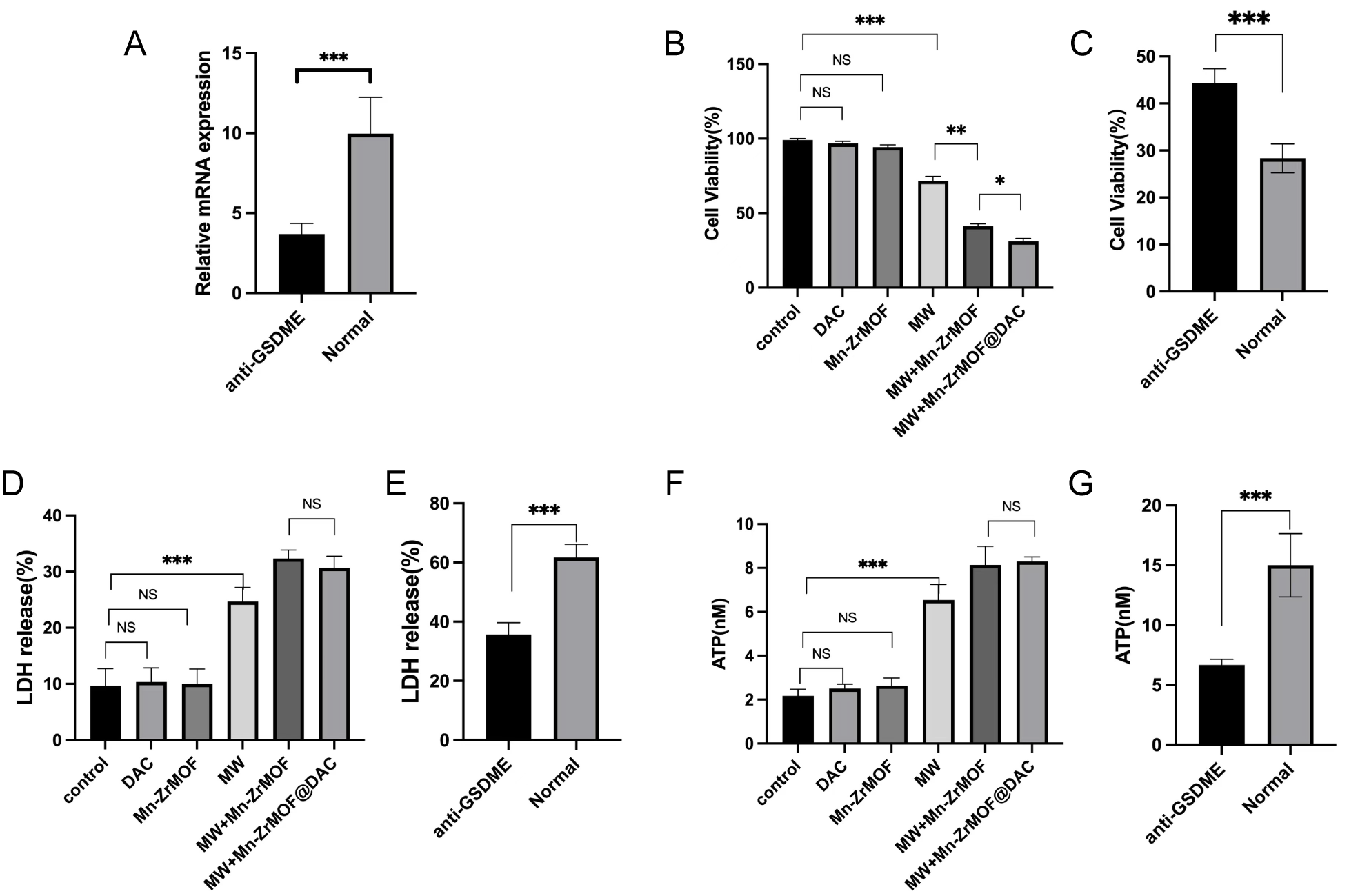

Supplement: Supplementary 1 — Figs. S1 to S5 Table S1 [file bmr.0077.f1.zip › S5.tif]
